# Mn_3_O_4_ nanozyme-based anti-inflammatory therapy modulates microglial phenotype by downregulating TLR4/NOX2 expression and further alleviates Alzheimer's disease pathology

**DOI:** 10.7150/thno.112213

**Published:** 2025-06-20

**Authors:** Jun Xie, Kai Cao, Luman Liu, Liding Zhang, Ying Yang, Hui Gong, Haiming Luo

**Affiliations:** 1 MOE Key Laboratory for Biomedical Photonics, Wuhan National Laboratory for Optoelectronics, Huazhong University of Science and Technology, Wuhan 430074, China.; 2 State Key Laboratory of Digital Medical Engineering, Key Laboratory of Biomedical Engineering of Hainan Province, School of Biomedical Engineering, Hainan University, Haikou 570228, China.; 3 Department of Pathophysiology, School of Basic Medicine, Key Laboratory of Education Ministry of China/Hubei Province for Neurological Disorders, Tongji Medical College, Huazhong University of Science and Technology, Wuhan 430074, China.; 4 HUST-Suzhou Institute for Brainsmatics, JITRI, Suzhou 215123, China.

**Keywords:** Alzheimer's disease, anti-inflammatory treatment, microglial phenotype, reactive oxygen species, nanozymes

## Abstract

**Rationale:** Evidence shows that neuroinflammation mediated by microglial activation plays an important role in Alzheimer's disease (AD) pathogenesis. However, the relationship between microglial phenotype and fibrillar β-amyloid (fAβ) pathology in anti-inflammatory treatment of AD remains unclear.

**Methods:** We designed a water-soluble Mn_3_O_4_ nanozymes and demonstrated its ability to reverse lipopolysaccharide (LPS)-induced microglial transition from M1 to M2 phenotype by clearing reactive oxygen species (ROS).

**Results:** In 5×FAD transgenic mice, intranasal (IN) instillation of Mn_3_O_4_ nanozymes initially promoted M2 microglial polarization and significantly reduced neuroinflammation after 4 weeks of treatment. After 8 weeks of continuous treatment, they further alleviate fAβ pathology and improved learning and memory deficits in 5×FAD mice. The excellent anti-inflammatory effect of Mn_3_O_4_ nanozymes is achieved by inhibiting the Toll-like receptor 4 (TLR4)/nicotinamide adenine dinucleotide phosphate (NAPDH) oxidase isoform 2 (NOX2) pathway to clear ROS.

**Conclusions:** This study reveals the molecular mechanism of Mn_3_O_4_ nanozymes modulating microglia phenotype to attenuate neuroinflammation primarily through inhibition of the TLR4/NOX2 pathway and highlights the temporal sequence of anti-inflammatory treatment in regulating microglial phenotype and improving fAβ pathology, providing new insights for the anti-inflammatory treatment of AD and other neurological diseases.

## Introduction

Alzheimer's disease (AD) is the most common cause of dementia, and its neuropathological characteristics include inflammation, intracellular neurofibrillary tangles (NFT), and amyloid plaque deposition of extracellular fibrillar β-amyloid protein (fAβ) 1. fAβ is formed by the aggregation of Aβ peptides, triggering an immune response and causing synaptic dysfunction, mitochondrial damage, microglial activation, and neuronal death 2, 3. Inflammation in AD is characterized by reactive microglia around Aβ plaques, which maintain an inflammatory state by secreting pro-inflammatory mediators, leading to neuronal loss 4. Based on the important role of neuroinflammation in AD pathology, reducing neuroinflammation has become a widely accepted and promising therapeutic strategy. Among them, reducing plasma Aβ, changing flora and reducing ROS are all considered to reduce neuroinflammation 5-7. In addition, studies have found a 50% reduction in the risk of developing AD in individuals with the use of nonsteroidal anti-inflammatory drugs, which emphasizes the importance of microglial activation-mediated neuroinflammation in AD 8. Inflammation plays an important role in AD pathogenesis, but its exact role is poorly understood.

Microglia are the main source of neuroinflammation because the expression and production of inflammatory cytokines by microglia are higher than those by other glial cells 9. Microglia are innate immune cells in the brain that participate in nerve development by phagocytosis and clearance of misfolded proteins and cell debris 10. However, once activated and displaying a pro-inflammatory phenotype, microglia can produce harmful substances in the brain. Microglia exhibit different phenotypes depending on the surrounding environment and can be roughly divided into classically activated (M1) and alternatively activated (M2) categories 11. Activation of the M1 phenotype leads to inflammation and neurotoxicity, whereas activation of the M2 phenotype triggers anti-inflammatory and repair responses 10. During the pathogenesis of AD, fAβ induces microglia to polarize toward the M1 phenotype, thereby releasing pro-inflammatory cytokines, nitric oxide (NO), and reactive oxygen species (ROS) 12. Excessive pro-inflammatory cytokines promote the production of Aβ, further stimulating the proliferation and activation of microglia 13. Thus, promoting the polarization of microglia to the M2 phenotype to alleviate neuroinflammation may be an effective strategy for treating AD, though it is challenging.

The transition of microglial phenotype is mainly affected by inflammation. Among them, toll-like receptors (TLRs), especially TLR4, can promote inflammatory response by activating downstream nuclear factor kappa-B (NF-κB), and promote microglia to polarize towards the M1 phenotype, thereby aggravating AD pathology 14. In addition, nicotinamide adenine dinucleotide phosphate (NAPDH) oxidase isoform 2 (NOX2), as the main source of ROS produced by microglia, is also considered to promote inflammatory response by activating NF-κB 15, 16. Accordingly, activation of the TLR4 receptor may promote NOX2 to produce a large amount of ROS, thereby exacerbating the inflammatory response and promoting the polarization of microglia to the M1 phenotype. Elimination of ROS by inhibiting the TLR4/NOX2 signaling pathway may effectively reduce inflammation and alleviate AD pathology.

Evidence shows that Aβ aggregates and damage-associated molecular patterns promote ROS production, thereby inducing oxidative stress 17. Sustained oxidative stress can damage functional proteins, lipids, and DNA, leading to extensive oxidative damage and neurodegeneration 18. Although there are many antioxidant enzymes naturally present in organisms, including superoxide dismutase (SOD), catalase (CAT), and glutathione peroxidase (GSH-Px), they are sensitive to the environment and may lose their bioactivity under pathological conditions 19, 20. With the development of nanotechnology, artificial nanozymes with the functions of mimicking multiple antioxidant enzymes have been developed to replace natural antioxidant enzymes, mainly including carbon-based, metal-based, and metal oxide-based nanozymes 21-23. Among them, metal oxide nanozymes have made breakthrough in disease diagnosis 24, 25, treatment 26-28, and biosensing 29-31 in recent years due to their high specific surface area, enzyme activity, and biocompatibility. Among metal oxide-based nanozymes, Mn_3_O_4_ nanozymes are widely used in anti-inflammatory treatment due to their antioxidant enzyme mimetic activity and excellent ROS scavenging effects. The enzyme mimetic activity of Mn_3_O_4_ nanozymes can inhibit a variety of peripheral inflammatory diseases *in vivo*, such as inflammatory bowel disease, liver injury, and osteoarthritis 32-34. In addition, Mn_3_O_4_ nanozymes have been shown to exerte redox effects, provide effective cellular protection, and improve cognitive function in Parkinson's disease model mice 35, 36. Although Mn_3_O_4_ nanozymes exhibit excellent ROS removal effects, whether Mn_3_O_4_ nanozymes can promote microglial polarization to M2 phenotype through TLR4/NOX2 and exert anti-inflammatory effects remains to be further studied.

**Scheme 1:**
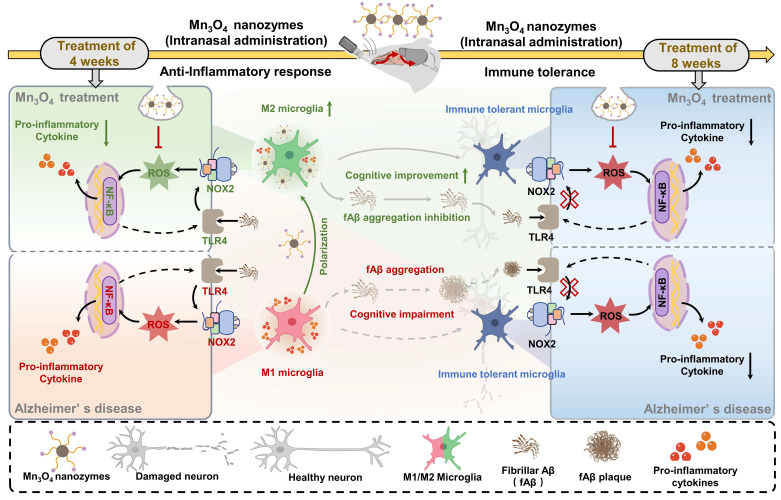
Illustration of the mechanism of Mn_3_O_4_ nanozymes regulating microglial phenotypes and further ameliorating AD pathology in 5×FAD mice: (1) The role of microglial polarization and TLR4/NOX2 in promoting neuroinflammation; (2) After 4 weeks of Mn_3_O_4_ nanozyme treatment, ROS generation was inhibited through the TLR4/NOX2 pathway, thereby regulating microglial phenotype; (3) Microglia exhibited immune tolerance, which attenuated the regulation of Mn_3_O_4_ nanozymes on the microglia phenotype of 5×FAD mice; (4) Mn_3_O_4_ nanozymes alleviated fAβ pathology and improved cognitive impairment of 5xFAD mice after 8 weeks of treatment.

In this study, a water-soluble antioxidant Mn_3_O_4_ nanozyme was synthesized and its molecular mechanism for regulating microglial phenotype was investigated (**Scheme 1**). The results showed that after 4 weeks of intranasal (IN) administration of Mn_3_O_4_ nanozymes, microglial phenotype was effectively modulated by downregulating TLR4/NOX2 expression. After 8 weeks, Mn_3_O_4_ nanozymes weakened the regulation of M2 microglial polarization but significantly improved fAβ pathology. This study clarifies the mechanism of antioxidant nanomaterials modulating microglial phenotypes, providing a new perspective for exploring the relationship between neuroinflammation and AD pathology.

## Materials and Methods

### Materials

Manganese acetate (Mn(Ac)_2_), oleic acid (OA), oleylamine, and bovine serum albumin (BSA) were purchased from Macklin Biochemical Co., Ltd (Shanghai, China); 1,2-distearoyl-sn-glycero-3-phosphoethanolamine-N-[Maleimide(polyethylene-glycol)-2000] (DSPE-PEG-Mal) and 1,2-distearoyl-sn-glycero-3-phosphoethanolamine-N-[amino(polyethylene-glycol)-2000] (DSPE-PEG- NH2) was ordered from Ponsure Biotechnology (Shanghai, China); SOD assay kit was purchased from Dojindo (Kumamoto, Japan); the CellTiter 96 Aqueous one solution cell proliferation assay was purchased from Promega (Madison, WI, USA); lipopolysaccharide (LPS) and 2',7'-Dichlorofluorescin diacetate (DCFH-DA) were purchased from Sigma-Aldrich (St. Louis, MO, USA); Iscove's modified dubecco's medium (IMDM) and fetal bovine serum (FBS) were purchased from Gibco BRL (Grand Island, NY, USA). Immunohistochemistry kits were purchased from Zhongshan Jinqiao Biotechnology Co., Ltd (Beijing, China); Radio immunoprecipitation assay (RIPA) lysis solution and 4',6-diamidino-2-phenylindole (DAPI) were purchased from BeyotimeBiotechnology Co., Ltd (Shanghai, China); Trizol reagent was purchased from Thermo Fisher Scientific (Shanghai, China); Enhanced chemiluminescence liquid (ECL), reverse transcription reagents, and Taq Pro Universal SYBR Master Mix were purchased from Vazyme (Nanjing, China); Phosphate buffer saline (PBS) was purchased from Biosharp (Beijing, China); Isoflurane was purchased from RWD Life science Co., Ltd (Shenzhen, China).

Rabbit monoclonal antibody to CD86 (19589S) was purchased from Cell Signaling Technology (Shanghai, China); rat monoclonal antibody to CD86 (14-0862-82) was purchased from Thermo Fisher Scientific (Shanghai, China); rabbit polyclonal antibody to CD206 mannose receptor (ab64693) and rabbit monoclonal antibody to NOX2/gp91phox (ab310337) were purchased from Abcam (Shanghai, China); rabbit polyclonal antibody to Iba-1 (019-19741) was purchased from Wako laboratory chemicals (Osaka, Japan); rabbit polyclonal antibody to NF-κB p65 (10745-1-AP) and rabbit monoclonal antibody to TLR4 (66350-1-Ig) were purchased from Proteintech (Wuhan, China); rabbit antibody to nuclear factor E2-related factor-2 (Nrf2) (T55136), rabbit antibody to Phospho-NF-κB p65 (TA2006), and mouse antibody to β-actin (T40104) were purchased from Abmart (Shanghai, China); rabbit polyclonal antibody to NeuN (GB11138), Alexa Fluor 488-labeled goat anti-rabbit IgG (GB25303), Cy3-labeled goat anti-rabbit IgG (GB21303), Cy3-labeled goat anti-rat IgG (GB21302), Cy5-labeled goat anti-rabbit IgG (GB27303), and HRP-conjugated goat anti-rabbit IgG (GB23303) and goat anti-mouse IgG (GB23301) were purchased from Servicebio (Wuhan, China). Fluor 647-conjugated anti-Aβ_42_ mouse mAb 2C6 was screened by our lab 37.

### Synthesis of Mn_3_O_4_ nanozymes

According to the reported method for the synthesis of Mn_3_O_4_ 38, 0.17 g Mn(Ac)_2_, 0.637 mL OA, and 3.28 mL oleylamine were dissolved in 15 mL xylene and heated to 90 °C. The mixture was vigorously stirred at 150 rpm, aged by adding 1 mL deionized water, and then maintained at 90 °C for 2.5 h. Mn_3_O_4_ nanozymes were obtained by adding 100 mL ethanol followed by centrifuging at 10,000 rpm for 10 min. To improve water solubility, Mn_3_O_4_ nanozymes were modified with DSPE-PEG-Mal groups 39. In brief, 1 mL of Mn_3_O_4_-cyclohexane was mixed with equal volume of ethanol and centrifuged at 10,000 rpm for 10 min. The pellet was dissolved in 1 mL chloroform and mixed with 25 mg DSPE-PEG-Mal dissolved in 2 mL chloroform. The solution was stirred at 150 rpm for 1 h and residual chloroform was removed using a nitrogen blower. The abovementioned dried sample was ultrasonically dispersed with 5 mL of PBS and centrifuged at 10,000 rpm for 10 min to obtain hydrophilic Mn_3_O_4_ nanozymes.

### Characterization of Mn_3_O_4_ nanozymes

The morphology and size of Mn_3_O_4_ nanozymes were determined using a transmission electron microscope (TEM; Tecnai G20, FEI Ltd., USA). The size distribution and Zeta potentials of Mn_3_O_4_ nanozymes were measured and analyzed using Zetasizer Nano (ZS90, Malvern Instruments, UK). The X-ray diffraction (XRD; PANalytical B.V., Holland) patterns of Mn_3_O_4_ nanozymes were used to analyze their structure and crystalline forms. The valence composition of Mn_3_O_4_ nanozymes was analyzed by energy-dispersive X-ray photoelectron spectroscopy (XPS; AXIS-ULTRA DLD-600W, Kratos, Japan). The SOD Assay Kit-WST was used to determine the SOD-like activity of Mn_3_O_4_ nanozymes according to the manufacturer's instructions. The SOD activity was measured with a microplate reader (BioTek, USA).

### Cell culture

The mouse N9 microglial cell line was purchased from Keycell Biotechnology Co., Ltd. (Wuhan, China), and cultured in IMDM medium containing 10% FBS, 2 mM L-glutamine, 100 μg/mL streptomycin, and 100 U/mL penicillin in an incubator at 37 °C with 5% CO_2_. Cells were subcultured at a split ratio of 1:4 and used for subsequent experiments.

### Cell viability

Cell viability was measured using a CellTiter 96 AQueous Assay. N9 microglia were seeded in 96-well plates at a density of 1×10^5^ cells/mL and cultured in a completed IMDM medium for 24 h. Subsequently, cells were treated with a completed IMDM medium containing different concentrations of Mn_3_O_4_ nanozymes. After 24 h of incubation, the culture medium was discarded, and the cells were incubated with fresh medium containing 20% CellTiter 96 Aqueous one solution at 37 °C for 3 h. Finally, the absorbance of each well was recorded at 450 nm using a microplate reader (BioTek, USA).

### Animals

The procedures for the care of animals and all animal experiments were approved by the Institutional Animal Care and Use Committee of Huazhong University of Science and Technology (IACUC Number: S2048). C57BL/6J mice (male, 6-8 weeks old) were purchased from the Hubei Experiment Animal Research Center (Wuhan, China). 5×FAD mice (male, 5-6 months old) were purchased from Beijing Huafukang Biotechnology Co., Ltd (Beijing, China). All animals were raised in standard ventilated cages, the temperature of the breeding room was controlled at 22 °C, and day and night alternated during feeding. Mn_3_O_4_ nanozymes were dissolved in PBS and intranasally instilled into C57BL/6J mice and 5×FAD mice three times a week at a dose of 2.4 mg/kg.

### Bilateral hippocampal injection of LPS

Mice were continuously anesthetized with isoflurane and fixed in a stereotactic apparatus. To maximize the stimulation of microglia polarization toward the M1 phenotype, LPS was injected into C57BL/6J mice via stereotactic injection at different doses of 10-200 μg. The coordinates were located on the bilateral hippocampus (Anterior-Posterior, -2.4 mm; Medial-Lateral, ±2.0 mm; Dorsal-Ventral, -2.2 mm from the bregma), and 2 μL of LPS was injected into one side of the hippocampus at a rate of 1 μL/min. The sham group experienced the same procedures with the same volume of saline. Animals were sacrificed on days 1, 3, 5, 7, and 14 after LPS injection. C57BL/6J mice were intranasally instilled with PBS or Mn_3_O_4_ nanozymes 1 h before stereotactic injection of LPS, and the treatment was continued for 7 days.

### Determination of intracellular ROS

The DCFH-DA fluorescent probe was used to evaluate the level of intracellular ROS in brain tissues 40. 5×FAD mice were treated with continuous intranasal instillation of Mn_3_O_4_ nanozymes for 4 or 8 weeks. Afterwards, cardiac perfusion was performed with PBS and the whole brain tissue was removed. The brain tissue was divided into two halves, one half of which was fixed with 4% paraformaldehyde overnight and sliced into 30 μm slices. The brain slices were incubated with DCFH-DA (10 μM) at 37 °C for 30 min. After rinsing with PBS, the slices were stained with DAPI, and the fluorescence signals were collected using a Zeiss LSM710 confocal microscope.

For the other half of the brain tissue, the hippocampus and prefrontal cortex were removed and homogenised with PBS. The extracted tissue supernatant was diluted with 10-fold volume of PBS and incubated with DCFH-DA (50 μM) at 37 °C for 30 min. Finally, the fluorescence absorbance of each well was recorded using a multifunctional microplate reader (Molecular Devices, USA).

### Immunohistochemistry (IHC)

CD86 and CD206 are considered as representative biomarkers of M1/M2 phenotype microglia 41. To detect the main phenotypes of microglia in the brains of bilateral hippocampus-injected LPS mice, brain tissues from C57BL/6J mice were collected and sectioned in the coronal plane. Antigen retrieval was performed on coronal slices of the brain tissue using the float method in citrate buffer. Sections were then stained according to the immunohistochemical kit. In brief, sections were incubated with 3% hydrogen peroxide for 20 min and goat serum for 1 h. Subsequently, primary antibodies CD86 (1:200, 19589S) and CD206 (1:1000) were added and the sections were incubated overnight at 4 °C. After fully washing with phosphate-buffered saline with tween 20 (PBST), the sections were incubated with biotinylated secondary antibody and horseradish peroxidase reaction solution at room temperature for 1 h. After incubation for 2 min with 3,3-diaminobenzidine, the sections were dehydrated and permeabilized with ethanol and xylene. Finally, the sections were observed and imaged via light microscopy (Nikon Eclipse Ni-E microscope, Japan). The positive areas were analyzed using the Image J software.

### Immunofluorescence (IF) imaging

Cellular IF staining was performed to detect the main microglial phenotypes after LPS stimulation. N9 microglia were seeded at a density of 5×10^4^ cells/well in a 24-well plate equipped with glass slides, cultured for 24 h, and then pre-treated with Mn_3_O_4_ nanozymes (1.1 μM) dispersed in complete IMDM medium for 2 h prior to LPS stimulation. Subsequently, the cells were stimulated with LPS (2 μg/mL) at select time points, and untreated cells were used as controls. The cells were then fixed with 4% paraformaldehyde for 20 min, blocked with 3% BSA for 1 h at room temperature, and incubated with primary antibodies including CD86 (1:200, 14-0862-82) and CD206 (1:1000) overnight at 4 °C. After washing three times with PBST, the cells were incubated with Cy3-labeled goat anti-rat IgG (1:500) or Alexa Fluor 488-labeled goat anti-rabbit IgG (1:500) in dark at room temperature for 1 h. Finally, the cells were imaged using a confocal microscope (LSM 710, Zeiss, Germany) and analyzed using Image J software.

Tissue immunofluorescence staining was used to detect the expression level of CD86, CD206, and NeuN in the prefrontal cortex and hippocampus of C57BL/6J and 5×FAD mice at the end of treatment. Coronal sections were first stained with primary antibodies such as CD86 (1:200, 14-0862-82), CD206 (1:1000), Iba-1 (microglia-specific marker, 1:500), NeuN (neuron-specific marker, 1:500), or Alexa anti-Aβ_42_ (fAβ composition) mouse mAb 2C6 (1:50) dispersed in 1% BSA and incubated overnight at 4°C. After washing with PBST, the sections were incubated with Cy3-labeled goat anti-rat IgG (1:500), Cy3-labeled goat anti-rabbit IgG (1:500) or Cy5-labeled goat anti-rabbit IgG (1:500 in dark at room temperature for 1 h. Finally, the sections were counterstained with DAPI. The fluorescence signals of these sections were acquired by a LSM710 confocal microscope (Zeiss, German).

### Cytokine assays

The cytokine levels were measured in the serum using the cytometric bead array (CBA) mouse inflammation kit (561665, BD Bioscience-Pharmingen, USA), allowing for simultaneous measurement of four anti-inflammatory factors and three pro-inflammatory factors in a single sample 7. The kit contains a detection antibody conjugated with phycoerythrin and seven bead populations coated with corresponding specific capture antibodies. These capture beads were incubated with recombinant standards or test samples to form sandwich complexes. Tests and analyses were performed according to the manufacturer's instructions. Briefly, blood was collected from the eyeballs of 5×FAD mice that had been intranasally injected with Mn_3_O_4_ nanozymes for 4 or 8 consecutive weeks, and the upper serum layer was obtained by centrifugation at 5000 rpm/min for 10 min. Further, 50 μL of premixed capture beads were mixed with 50 μL of PE detection reagent. After adding 50 μL of the supplied standards or serum samples, the mixture was incubated in dark at room temperature for 3 h. The mixture was then washed and centrifuged at 200 g for 5 min. Finally, the pellet was resuspended in 300 μL of wash buffer. The complex was separated in the FL-3 channel of a FACSCalibur flow cytometer (BD Bioscience-Pharmingen, USA), and the corresponding standard reference curve was constructed using the CBA analysis software (BD Bioscience-Pharmingen, USA).

### Y-maze

After 8 weeks of PBS or Mn_3_O_4_ nanozyme treatment, the 5×FAD mice were subjected to the Y-maze test using a device with three equiangular white arms. The mice were placed on the end of one arm and explored freely in the maze for 8 min. The number of arm entries and alternations was manually recorded. Spontaneous alteration behavior (%) = actual alterations/total number of arm entries.

### Morris water maze (MWM)

After 8 weeks of PBS or Mn_3_O_4_ nanozyme treatment, 5×FAD mice were scheduled for the MWM test to assess their spatial learning and memory ability. The experimental device was a circular pool with a 120 cm diameter, and the water temperature was maintained at 19-22 °C. The pool was equally divided into four quadrants, and a platform with a height of 1 cm below the water surface was placed in one of the quadrants. In the place navigation stage, the training was performed 4 times per day for a total of 5 days. During the training process, the mice were put into the pool from different quadrants facing the pool wall for 60 s, and the time to find the platform was recorded. The mice that could not find the platform were placed on the platform for 30 s. In the space exploration stage on the sixth day, the mice were put into the water pool from the other side of the original platform for 60 s, and the times of crossing the original platform were recorded. The trajectory of mice was recorded using a camera and analyzed by EthoVision XT 8.0.

### Quantitative real-time polymerase chain reaction (RT-qPCR)

The mRNA expression levels of NOX2, Nrf2, and TLR4 in the hippocampus and prefrontal cortex were analyzed by RT-qPCR. After 4 and 8 weeks of PBS or Mn_3_O_4_ nanozymes treatment, the total RNA from brain tissues of 5×FAD mice was isolated using a Trizol reagent according to the manufacturer's instructions. Total RNA (1.0 μg) was reverse transcribed to cDNA using reverse transcription reagents. RT-PCR analysis of NOX2, Nrf2, and TLR4 mRNA was performed using the QuantStudio 3 RT-PCR system (Applied Biosystems; Thermo Fisher Scientific, Waltham, MA, USA) with SYBR qPCR master mix. All operations were performed following the manufacturer's instructions. Changes in mRNA levels were determined with the help of normalized internal controls (β-actin mRNA). The primer sequences used for RT-qPCR were as follows: NOX2 (F-TCGCTGGAAACCCTCCTATG, R-GGATACCTTGGGGCACTTGA); Nrf2 (F-CCCAGCACATCCAGACAGAC, R-TATCCAGGGCAAGCGACTCA); TLR4 (F-AACTTCAGTGGCTGGATT, R-ACTAGGTTCGTCAGATTGG); β-actin (F-GTGCTATGTTGCTCTAGACTTCG, R- ATGCCACAGGATTCCATACC).

### Western blotting (WB)

All hippocampus and prefrontal cortex tissues of 5×FAD mice and the hippocampus of C57BL/6J mice were collected and homogenized in RIPA lysis buffer containing protease inhibitors for 30 min. The lysates were centrifuged at 12,000 rpm for 15 min at 4 °C, and the total protein concentration in the supernatant was measured using a BCA protein assay kit. Equal amounts of proteins were separated on 10-12% sodium dodecyl sulfate-polyacrylamide gel electrophoresis and transferred to polyvinylidene fluoride membranes. The membranes were blocked in 5% skimmed milk for 1 h at 37 °C. Subsequently, primary antibodies including CD86 (1:200, 19589S), CD206 (1:1000), NOX2 (1:1000), NF-κB p65 (1:2000), TLR4 (1:2000), Nrf2 (1:1000), Phospho-NF-κB p65 (1:1000), and β-actin (1:1000) were incubated overnight at 4 °C. After washing with PBST, the membranes were incubated with the corresponding HRP-conjugated second antibody (1:5000) at 37 °C for 1 h. Immunoreactive bands were visualized using ECL and analyzed using Image J software.

### Statistical analysis

All statistical analyses were performed using GraphPad Prism 9.0 software, and the results are presented as mean ± standard error of mean (SEM). Differences between two groups were compared using unpaired t-test or multiple t-tests. For additional data on multigroup comparisons, one-way or two-way analysis of variance were performed, followed by Tukey's test or post-hoc Bonferroni's multiple comparison test to compare the differences. Statistical significance is indicated in the figure with ^*^p < 0.05, ^**^p < 0.01, ^***^p < 0.001, and ^****^p < 0.0001, whereas ns indicates no significance.

## Results

### Mn_3_O_4_ nanozymes modulated the M1/M2 phenotype of LPS-treated N9 microglia

**Figure 1:**
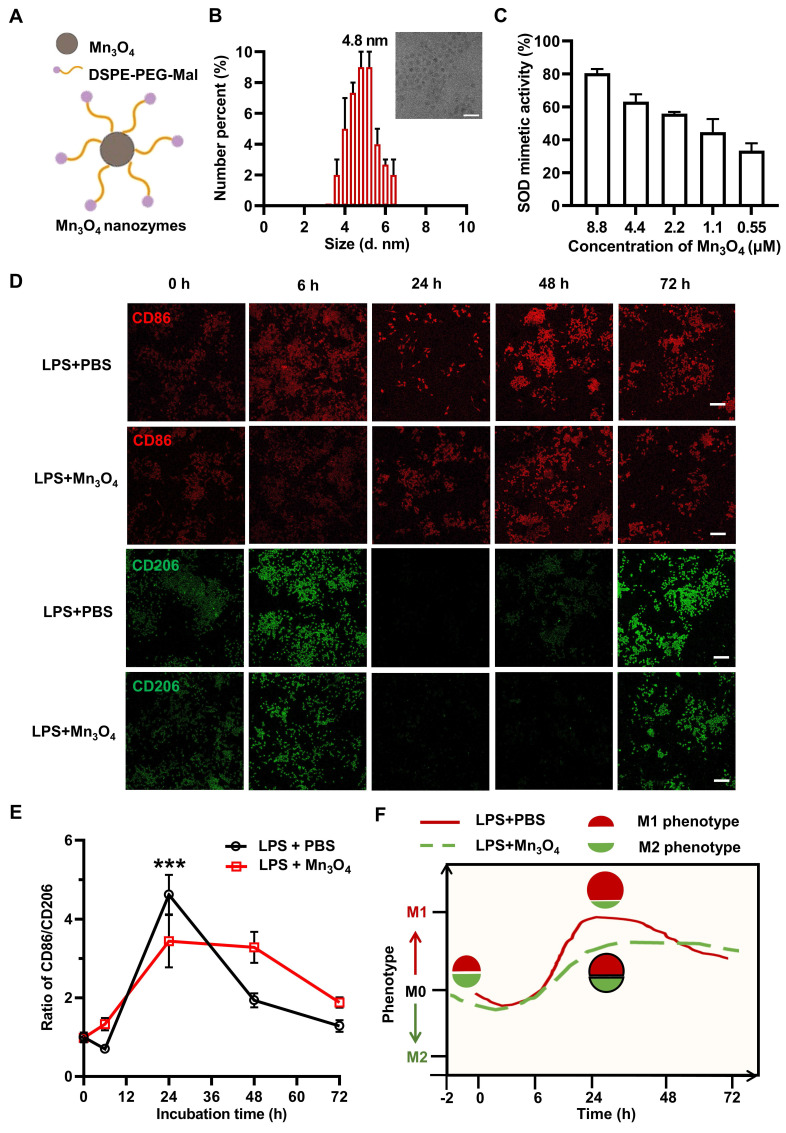
** Modulatory effect of Mn_3_O_4_ nanozymes on the expression of M1 and M2 markers in N9 microglia under LPS stimulation. (A)** Schematic representation of Mn_3_O_4_ nanozymes. **(B)** TEM image of Mn_3_O_4_ nanozymes and average size of Mn_3_O_4_ nanozymes measured by DLS, scale bar = 20 nm. **(C)** SOD-like activity of Mn_3_O_4_ nanozymes (0.55-8.8 μM). **(D-E)** Representative immunofluorescence images and statistical analysis of the ratio of CD86 to CD206 in LPS-induced (2 μg/mL) N9 microglia after treatment with Mn_3_O_4_ nanozymes (1.1 μM) at different time points (0-72 h), scale bar = 100 μm. **(F)** Schematic diagram of Mn_3_O_4_ nanozyme regulating the M1/M2 phenotype of LPS-treated N9 microglia. Data are presented as mean ± SEM, n = 3. Multiple t-tests were used for multigroup comparison.^ ***^p < 0.001.

The structure and synthesis of Mn_3_O_4_ nanozyme are shown in **Figure 1A** and **Figure S1A**. Mn_3_O_4_ nanozyme was successfully synthesised by hydrothermal method, and crystal planes such as (2 1 1), (2 2 0), and (2 2 4) were detected, indicating its hausmannite structure (**Figure S1B**). After DSPE-PEG-Mal modification, Mn_3_O_4_ nanozyme was transferred from oil phase to aqueous phase, and the zeta potential of the nanozyme decreased from ~0 mV to ~-5 mV (**Figure S1D**), which is consistent with the previous results of DSPE-PEG-Mal modified material 42. In addition, TEM revealed that the particle size of Mn_3_O_4_ nanozyme was uniform, with a diameter of 4.8 nm (**Figure 1B**). The aqueous kinetic diameter reached about 100 nm (**Figure S2B**), which may be due to the distribution of DSPE-PEG-Mal micelles around the Mn_3_O_4_ nanozymes. In addition, we further explored the various antioxidant enzyme activities and free radical scavenging abilities of Mn_3_O_4_ nanozymes. The results showed that the antioxidant enzyme mimetic activity of Mn_3_O_4_ nanozymes was mainly manifested as SOD-like enzyme activity, while the specific enzyme activity against CAT and GSH-Px was low (**Figure S3A**). Also, Mn_3_O_4_ nanozymes also exhibited good scavenging activity against superoxide anions (·O_2_^-^) and hydroxyl radicals (·OH) (**Figure 1C and Figure S3**), which is consistent with the XPS results showing the simultaneous presence of Mn(II) and Mn(III) in the Mn_3_O_4_ nanozyme (**Figure S1C**).

The phenotypic shift of microglia is mainly affected by inflammatory responses 43. Therefore, the classic pro-inflammatory agent LPS was used to induce the phenotypic transition of microglia. To determine the optimal LPS concentration for inducing the M1 polarization of N9 microglia, cells were treated with 0, 0.5, 2, 4, and 10 μg/mL of LPS for 24 h. The expression levels of the pro-inflammatory M1 marker CD86 and the anti-inflammatory M2 marker CD206 were then analyzed. The results showed that 2 μg/mL LPS significantly enhanced the M1 polarization of N9 microglia (**Figure S4**). Therefore, 2 μg/mL LPS was selected as an inducer to establish a cell model for evaluating the regulatory effect of Mn_3_O_4_ nanozymes on microglial phenotype.

To confirm the regulatory effects of Mn_3_O_4_ nanozymes on microglial phenotype, we first evaluated their cytotoxicity in N9 cells. Cell viability results showed that Mn_3_O_4_ nanozymes had negligible toxicity at concentrations below 1.1 μM (**Figure S5A**). Therefore, 0.55 μM and 1.1 μM of Mn_3_O_4_ nanozymes were selected for subsequent experiments. N9 cells were pretreated with 0.55 μM and 1.1 μM of Mn_3_O_4_ nanozymes for 2 h and exposed to 2 μg/mL of LPS for 24 h. Immunofluorescence analysis showed that the CD86/CD206 fluorescence intensity ratio in N9 microglia significantly increased after LPS treatment (*p* < 0.01), indicating a shift toward M1 polarization. In contrast, pretreatment with Mn_3_O_4_ nanozymes significantly reduced this ratio (*p* < 0.01, **Figure S5B-C**), indicating that Mn_3_O_4_ nanozymes effectively inhibited M1 polarization in N9 microglia.

To further investigate the effects of Mn_3_O_4_ nanozymes on microglial phenotypes, we monitored their dynamic changes for 6 to 72 h. Under normal culture conditions, N9 microglia showed weak fluorescence signals for CD86 and CD206. After 6 h of LPS stimulation of N9 microglia, the fluorescence intensities of CD86 and CD206 showed an increasing trend. Afterwards, the CD86 signal persisted even after 72 h of LPS stimulation, while the CD206 signal weakened after 24-48 h of LPS stimulation of N9 microglia. Notably, although the fluorescence intensity ratio of CD86/CD206 increased significantly to 4.3 at 24 h of LPS stimulation, pretreatment with 1.1 μM Mn_3_O_4_ nanozyme significantly reduced the fluorescence intensity of CD86/CD206 to 3.7 (**Figure 1D-E**). These results confirmed that N9 microglia shifted from a quiescent state to an M1 phenotype after 24 h of LPS stimulation, and that Mn_3_O_4_ nanozyme inhibited the polarization of N9 microglia toward an M1 phenotype after prolonged LPS stimulation for 24 h (**Figure 1F**).

### Mn_3_O_4_ nanozymes regulated microglial polarization by inhibiting TLR4 expression

To determine the optimal LPS concentration for inducing M1 polarization of hippocampal microglia in the mouse model, we injected different doses of LPS directly into the hippocampus and monitored changes in microglial polarization 7 days after LPS injection. Immunohistochemistry results showed that a dose of 80 μg of LPS resulted in the highest CD86/CD206 ratio (*p* < 0.05), indicating that the polarization of microglia to the M1 phenotype was most obvious at this concentration (**Figure S6 and S7**). Therefore, a neuroinflammation model to evaluate the regulatory effect of Mn_3_O_4_ nanozymes on microglial polarization was established with the help of a single injection (80 μg) of LPS into the hippocampus over 7 days.

**Figure 2:**
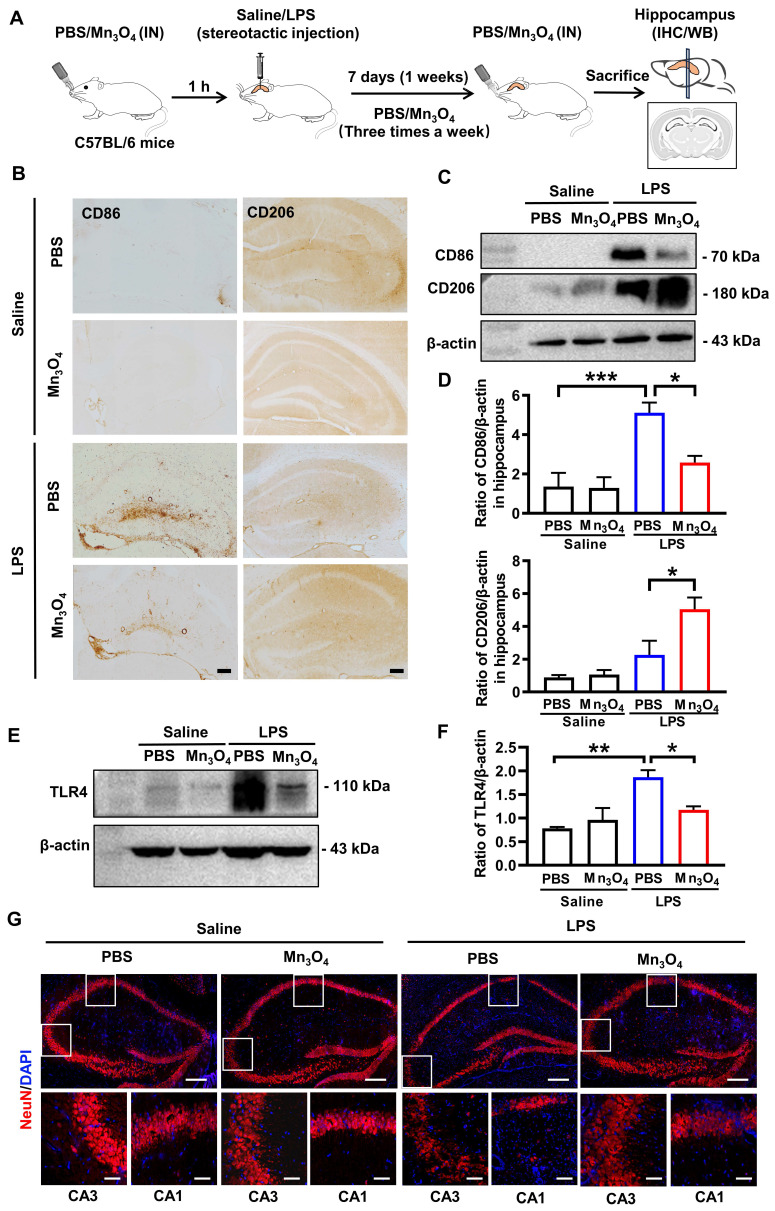
** Mn_3_O_4_ nanozymes modulated M2 microglial polarization by inhibiting TLR4 expression after bilateral hippocampal injection of LPS in C57BL/6J mice. (A)** Schematic diagram of the experimental design in C57BL/6J mice. **(B)** Representative immunohistochemical images of CD86 and CD206 in the hippocampus of LPS-treated mice after treatment with Mn_3_O_4_ nanozymes, scale bar = 100 μm. **(C)** Western blotting analysis of proteins involved in CD86/CD206 in the hippocampus of LPS-treated mice after treatment with Mn_3_O_4_ nanozymes.** (D)** Statistical analysis of CD86 and CD206 protein expression levels after the above treatments. **(E)** Representative western blotting of TLR4 protein in the hippocampus of LPS-treated mice after treatment with Mn_3_O_4_ nanozymes. **(F)** Statistical analysis of TLR4 receptor after the abovementioned treatments. **(G)** Representative immunofluorescence staining of NeuN-positive neurons in the hippocampus of LPS-treated mice after the corresponding treatments, scale bar = 200 μm. Magnified images of the boxed areas of CA1 and CA3 brain regions are shown below the full images, scale bar = 50 μm. Data are presented as mean ± SEM, n = 3. ANOVA was used for multigroup comparisons. ^***^p < 0.001, ^**^p < 0.01, ^*^p < 0.05.

Subsequently, to investigate whether Mn_3_O_4_ nanozymes modulate LPS-induced microglial polarization, we administered Mn_3_O_4_ nanozymes intranasally into mice that had been pretreated with a single dose of LPS (80 μg) in hippocampus (**Figure 2A**). Before investigating the regulatory effect of Mn_3_O_4_ nanozymes on the phenotype of brain microglia, we first verified the metabolism and distribution of Mn_3_O_4_ nanozymes in the brain and major peripheral organs. We dripped Cy3-labeled Mn_3_O_4_ nanozymes into the nasal cavity of mice and performed tissue fluorescence imaging at different times. The results showed that fluorescent signals could be observed in the brain after 1-3 h of intranasal administration of Mn_3_O_4_ nanozymes (**Figure S8**), and further distributed to the brain, liver, and kidney within 6 h, and almost completely eliminated from the body after 36 h (**Figure S9**). This indicates that nasally instilled Mn_3_O_4_ nanozymes can enter the brain without the risk of accumulation in the body. Afterwards, we further observed the co-localization of Cy3-labelled Mn_3_O_4_ nanozymes with different neuronal cells by nasal instillation. Immunostaining results showed that 6 h after nasal instillation, Cy3-labeled Mn_3_O_4_ nanozyme showed co-staining of Cy3 with NeuN neurons and Iba-1 microglia in the mouse hippocampus (**Figure S10**). This indicates that Mn_3_O_4_ nanozymes are not specific to microglia and can enter other neuronal cells in brain tissue.

In addition, we further explored the biocompatibility of Mn_3_O_4_ nanozymes. The results of the hemolysis experiment showed that when the concentration of Mn_3_O_4_ nanozymes was 1 mg/mL, Mn_3_O_4_ nanozymes did not cause obvious hemolysis even after 24 h (**Figure S11**). At the same time, we performed blood biochemical analysis on healthy mice injected with Mn_3_O_4_ nanozymes intranasally for 24 h. The results showed that the biochemical parameters of serum alanine aminotransferase (ALT), aspartate aminotransferase (AST), blood urea nitrogen (BUN), and creatinine (CREA) were all within the normal range 44, indicating that Mn_3_O_4_ nanozymes do not cause significant acute damage to liver and kidney function *in vivo* (**Figure S12**). Thus, the above results collectively indicate that Mn_3_O_4_ nanozymes are biocompatible and do not cause significant acute damage to liver and kidney functions *in vivo*.

For the regulation of Mn_3_O_4_ nanozymes on microglia and anti-inflammatory effects, immunohistochemistry and western blotting analysis showed that LPS treatment significantly enhanced the expression of CD86 in the hippocampus compared with the control group (*p* < 0.001), indicating that LPS promotes microglial polarization to the M1 phenotype. In contrast, after treatment with Mn_3_O_4_ nanozymes, the CD86 expression level was significantly decreased, whereas that of CD206 was significantly increased (*p* < 0.05, **Figure 2B-D and Figure S13A-B**), indicating that Mn_3_O_4_ nanozymes promote the transition of microglia from the M1 pro-inflammatory phenotype to the M2 anti-inflammatory phenotype. TLR4 is a key receptor controlling M1 polarization on the microglia membrane 45. Therefore, we further investigated whether Mn_3_O_4_ nanozymes would affect TLR4 expression in the hippocampus of LPS-treated mice. Western blotting results showed that LPS significantly increased TLR4 expression in the microglia in the hippocampus of C57BL/6J mice (*p* < 0.01), whereas Mn_3_O_4_ nanozyme treatment significantly alleviated this upregulation (*p* < 0.05, **Figure 2E-F and Figure S13C**). These findings suggest that Mn_3_O_4_ nanozymes exert anti-inflammatory effects by attenuating LPS-induced upregulation of TLR4 in M1 microglia.

Finally, we quantified the expression level of NeuN protein in neurons located in the hippocampal region to evaluate whether Mn_3_O_4_ nanozymes can alleviate LPS-induced neuronal injury in mice by regulating microglial polarization. Immunofluorescence analysis showed that the area of NeuN-positive neurons in the CA1 (*p* < 0.01) and CA3 (*p* < 0.001) regions of the hippocampus was significantly reduced in LPS-treated mice compared with that in control mice. However, after treatment with Mn_3_O_4_ nanozymes, the area of NeuN-positive neurons in both (*p* < 0.01 and *p* < 0.001) regions was significantly increased (**Figure 2G and Figure S14**), indicating that Mn_3_O_4_ nanozymes can significantly alleviate LPS-induced neuronal injury. Therefore, our results indicate that Mn_3_O_4_ nanozymes can not only inhibit LPS-induced TLR4 upregulation, but also promote the transition of microglia from M1 to M2 phenotype, thus highlighting its neuroprotective potential and therapeutic applicability in neurodegenerative diseases.

### Mn_3_O_4_ nanozymes mainly affected microglial phenotype rather than fAβ pathology in 5×FAD mice after 4 weeks of treatment

**Figure 3:**
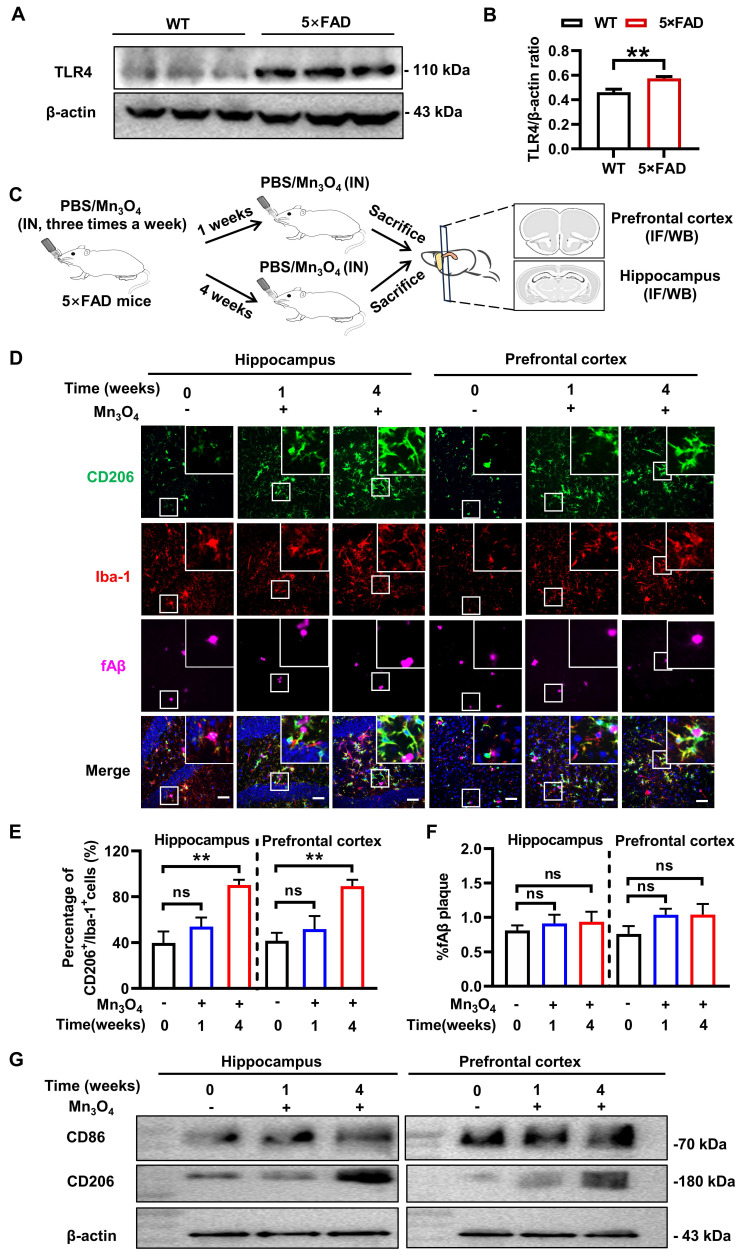
** Modulation of microglial phenotype and its effect on fAβ plaques after 1-4 weeks of treatment of 5×FAD mice with Mn_3_O_4_ nanozymes. (A)** TLR4 expression levels in the prefrontal cortex and hippocampus of WT and 5×FAD mice as measured by western blotting. **(B)** Comparison of the ratio of TLR4/β-actin in the prefrontal cortex and hippocampus of wild-type and 5×FAD mice, n = 6. **(C)** Schematic diagram of the experimental scheme for 1-4 weeks of Mn_3_O_4_ nanozyme treatment in 5×FAD mice. **(D)** Immunofluorescence staining results of CD206 (green), Iba-1 (red), and fAβ plaques (Magenta) in the hippocampus and prefrontal cortex of 5×FAD mice treated with Mn_3_O_4_ nanozymes for 1-4 weeks, scale bar = 50 μm. The upper right corner of each image is an enlarged view of the boxed area. **(E)** Statistics of the percentage of CD206^+^/Iba-1^+^ cells in the enlarged images of the hippocampus and prefrontal cortex, n = 6. **(F)** Statistics of the percentage of Aβ-positive areas in the hippocampus and prefrontal cortex of 5×FAD mice after the indicated treatments, n = 3.** (G)** Immunoblotting results of CD86 and CD206 expression levels in the hippocampus and prefrontal cortex tissues after 1-4 weeks of treatment with Mn_3_O_4_ nanozymes. Data are presented as mean ± SEM. Unpaired t-test was used for two-group comparisons and ANOVA was used for multigroup comparisons. ^**^p < 0.01, and ns (no significance).

The pathological protein fAβ is a pathogenic factor of AD and a ligand that activates TLR4 receptors on the microglial membrane 46. TLR4 receptor activation triggers an inflammatory response and promotes microglial polarization toward the M1 phenotype 47. To verify this phenomenon, we performed a comparative analysis of TLR4 protein expression in the prefrontal cortex and hippocampus of wild-type (WT) and 5×FAD transgenic mice. The results showed that TLR4 expression level was significantly upregulated in 5×FAD transgenic mice (*p* < 0.01, **Figure 3A-B and Figure S15A**). Given that Mn_3_O_4_ nanozymes inhibit LPS-induced TLR4 upregulation and promote microglial polarization towards the M2 phenotype, we further explored the potential of Mn_3_O_4_ nanozymes to modulate microglial phenotype and alleviate the pathology in 5×FAD mice.

The treatment scheme of Mn_3_O_4_ nanozyme for 5×FAD mice is shown in **Figure 3C**. Since 5×FAD mice need to be continuously nasally administered with Mn_3_O_4_ nanozymes, we first evaluated the stability of the nanozymes. The results showed that after incubation at 37 °C for 28 days, the particle size and potential of the Mn_3_O_4_ nanozyme did not change significantly (**Figure S2**). In addition, we further evaluated the release of manganese ions from Mn_3_O_4_ nanozymes in the nasal environment by inductively coupled plasma-optical emission spectroscopy (ICP-OES) analysis in a simulated nasal electrolyte solution (pH = 5.5). The results showed that the release of manganese ions from Mn_3_O_4_ nanozymes could last for 28 days, and the final release rate was less than 15%, which also indicated that Mn_3_O_4_ nanozymes had good stability in nasal environment (**Figure S16**). In addition, we further examined the long-term antioxidant capacity of Mn_3_O_4_ nanozymes. The results showed that after incubation at 37 °C *in vitro* for 28 days, Mn_3_O_4_ nanozymes still maintained good ·O_2_^-^ and ·OH scavenging capacity. In particular, compared with the control group, the ·O_2_^-^ scavenging rate of Mn_3_O_4_ nanozymes did not decrease significantly after incubation at 37 °C *in vitro* for 28 days (**Figure S17**). The above results indicate that Mn_3_O_4_ nanozymes have good stability and long-term antioxidant capacity *in vitro*. However, the SOD specific enzyme activity and ·O_2_^-^ scavenging rate of Mn_3_O_4_ nanozymes were significantly lower than those of natural SOD enzymes (**Figure S18**). Considering that natural SOD enzymes are easily inactivated and rapidly consumed *in vivo* 48, Mn_3_O_4_ nanozymes can replace natural antioxidant enzymes to exert long-term antioxidant effects *in vivo* due to their excellent stability.

Subsequently, we monitored the changes in microglia phenotype by immunofluorescence after 1 and 4 weeks of Mn_3_O_4_ nanozyme treatment.The results showed that after 1 week of treatment, the percentage of CD86^+^/Iba-1^+^ and CD206^+^/Iba-1^+^ microglia in the hippocampus and prefrontal cortex did not change significantly (**Figure 3D-E, Figure S19A-B**). After 4 weeks of treatment, the percentage of CD206^+^/Iba-1^+^ microglia increased significantly (*p* < 0.01,** Figure 3D-E**), but the percentage of CD86^+^/Iba-1^+^ microglia remained unchanged (**Figure S19A-B**). Western blotting results showed that CD86 and CD206 protein expression levels were consistent with the results of immunofluorescence analysis (*p* < 0.01, **Figure 3G, Figure S15B-E and S19C-D**). In addition, immunofluorescence analysis showed no significant change in the positive area of fAβ plaques in the hippocampus and prefrontal cortex after 1-4 weeks of treatment (**Figure 3D and F**). These results indicated that after 4 weeks of Mn_3_O_4_ nanozyme treatment, the number of M2 microglia in the brains of 5×FAD mice increased significantly, but fAβ pathology was not significantly improved.

### Mn_3_O_4_ nanozymes improved fAβ pathology and cognitive memory in 5×FAD mice after 8 weeks of treatment

**Figure 4:**
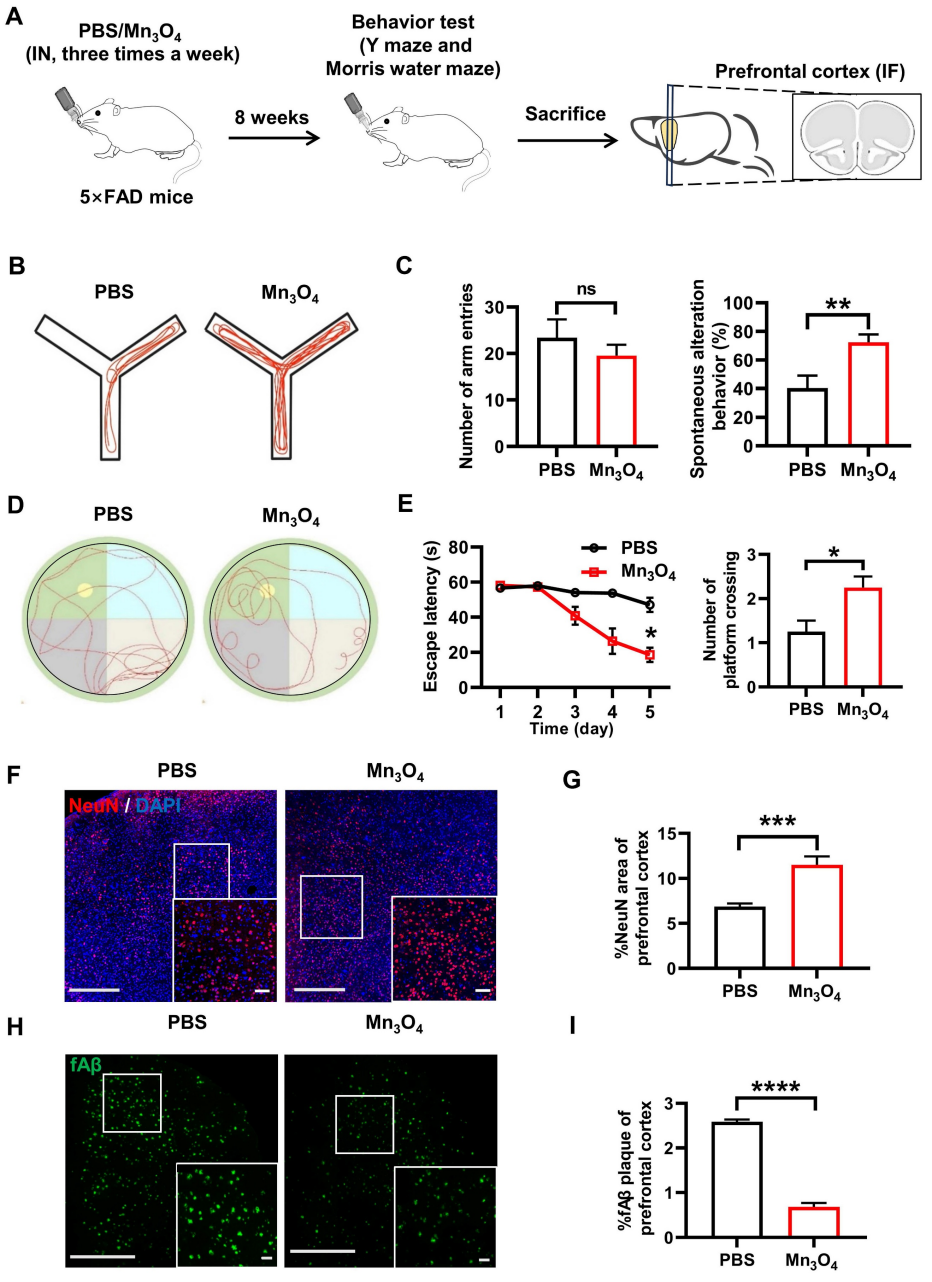
** Mn_3_O_4_ nanozyme treatment for 8 weeks alleviated fAβ pathology and improved cognitive function in 5×FAD mice. (A)** Schematic representation of the 8-week treatment regimen with Mn_3_O_4_ nanozymes in 5×FAD mice. **(B)** Schematic diagram of the movement route of 5×FAD mice in the Y-maze experiment after 8 weeks of Mn_3_O_4_ nanozyme treatment. **(C)** Statistics of the number of entering different arms and the spontaneous alternation rate in the Y-maze, n = 7 per group. **(D)** Movement routes of 5×FAD mice in the probe phase of the MWM experiment after 8 weeks of treatment. **(E)** Statistics of escape latency and platform crossing times in the MWM experiment, n = 4 per group. **(F)** Immunofluorescence staining of NeuN in the prefrontal cortex of 5×FAD mice treated with Mn_3_O_4_ nanozymes for 8 weeks, scale bar = 100 μm; magnified images, scale bar = 50 μm. **(G)** Statistical data showing the magnified images of NeuN-positive areas in the prefrontal cortex, n = 6 per group. **(H)** Immunofluorescence staining and magnified images of fAβ plaques in the prefrontal cortex of 5×FAD mice treated with Mn_3_O_4_ nanozymes for 8 weeks, scale bar = 200 μm; magnified images, scale bar = 50 μm. **(I)** Statistical data showing the magnified images of positive areas of fAβ plaques in the prefrontal cortex, n = 3 per group. Data are presented as mean ± SEM. Unpaired t-test or two-way ANOVA was used for two-group comparisons.^ ****^p < 0.0001, ^***^p < 0.001, ^**^p < 0.01, ^*^p < 0.05, and ns (no significance).

We further extended the treatment time to 8 weeks to evaluate the potential of Mn_3_O_4_ nanozymes to improve AD pathology and enhance cognitive function (**Figure 4A**). We first evaluated the biosafety of continuous intranasal administration of Mn_3_O_4_ nanozymes for 8 weeks. The results of mouse body weight showed that there was no significant difference between the group treated with nasal administration of Mn_3_O_4_ nanozymes and the PBS-treated group (**Figure S20A**). In addition, hematoxylin and eosin (H&E) staining results also showed no obvious cell necrosis and inflammation in all organs after 8 weeks of intranasal administration of Mn_3_O_4_ nanozymes (**Figure S20B**). H&E staining of nasal mucosal tissues showed that compared with the PBS-treated group, Mn_3_O_4_ nanozymes did not cause inflammation and damage to the nasal mucosa after long-term intranasal administration (**Figure S21**). These results indicate that Mn_3_O_4_ nanozymes exhibit good biosafety and can be used for long-term intranasal administration against AD.

Subsequently, we further investigated the changes in cognitive function and AD pathology in 5×FAD mice after 8 weeks of continuous intranasal administration of Mn_3_O_4_ nanozymes. The Y-maze test results showed that the spontaneous alternation ability of 5×FAD mice was significantly enhanced after 8 weeks of Mn_3_O_4_ nanozyme treatment (*p* < 0.01, **Figure 4B-C**). Notably, there was no significant difference in the number of arm entries compared with PBS-treated mice, indicating that Mn_3_O_4_ nanozymes effectively enhanced the working memory of 5×FAD mice. The MWM test results showed that the escape latency of mice in the Mn_3_O_4_ was shorter than that of PBS-treated mice (*p* < 0.05). In addition, the number of platform crossings in the exploration phase significantly increased (*p* < 0.05, **Figure 4D-E**), suggesting that Mn_3_O_4_ nanozymes enhance the spatial learning and memory of 5×FAD mice.

Immunofluorescence analysis results further showed that Mn_3_O_4_ nanozymes significantly increased the NeuN-positive area of prefrontal cortical neurons (*p* < 0.001, **Figure 4F-G**) and significantly reduced the positive area of fAβ plaques (*p* < 0.0001, **Figure 4H-I**), demonstrating its efficacy in improving AD pathology. These results suggest that Mn_3_O_4_ nanozymes can significantly alleviate AD pathological progression and cognitive impairment in 5×FAD mice by promoting microglial polarization to the M2 phenotype.

### Mn_3_O_4_ nanozymes inhibited inflammation and promoted modulation of microglial phenotype during 4 weeks of treatment

**Figure 5:**
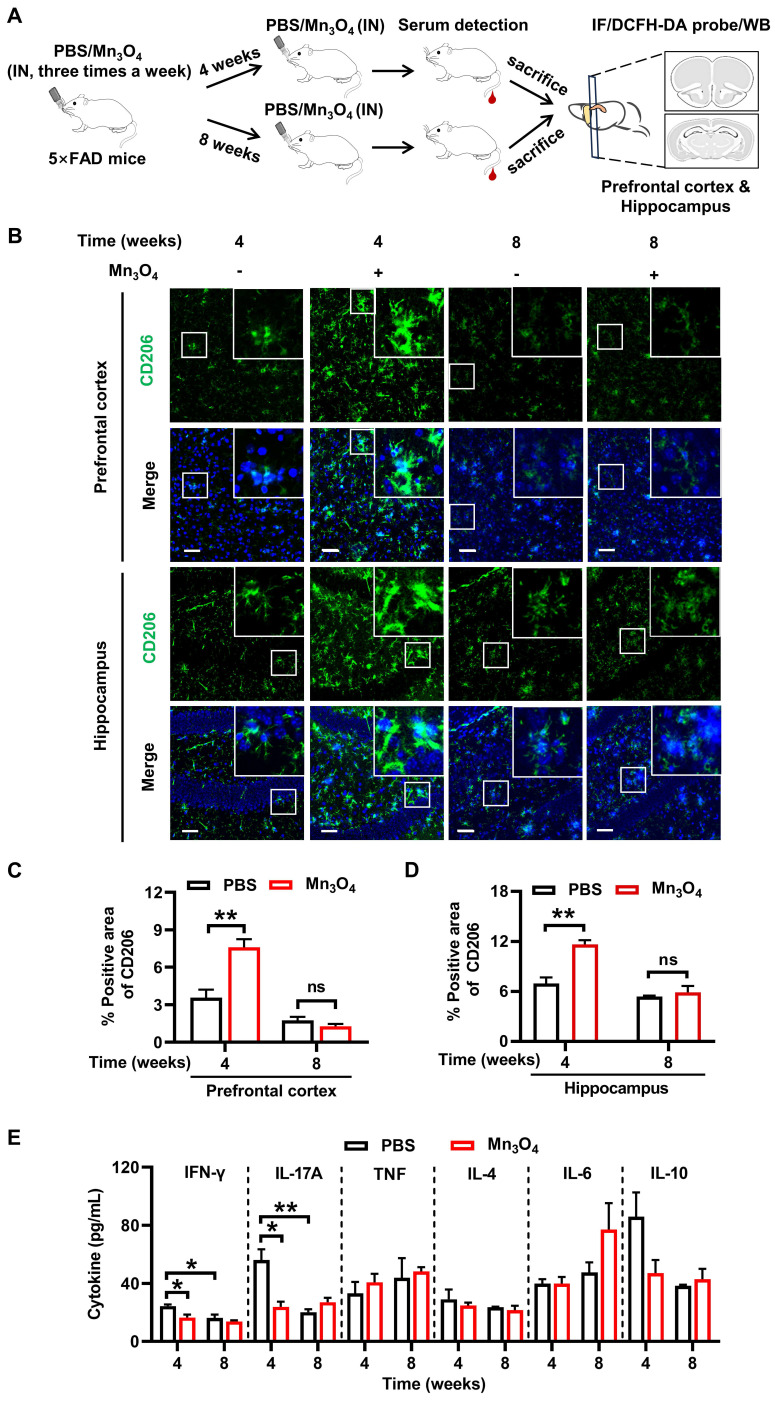
** Mn_3_O_4_ nanozymes promoted microglial polarization toward M2 phenotype in 5×FAD mice by inhibiting inflammatory response. (A)** Schematic diagram of the protocol for 4-8 weeks of Mn_3_O_4_ nanozyme treatment regimen in 5×FAD mice. **(B)** Immunofluorescence staining of CD206 (green) in the prefrontal cortex and hippocampus of 5×FAD mice after 4 and 8 weeks of Mn_3_O_4_ treatment, scale bar = 50 μm. Magnified images of the boxed areas are shown in the upper right corner of each image.** (C-D)** Statistical analysis of the percentage of CD206-positive areas in the prefrontal cortex and hippocampus after the indicated treatments, n = 3 per group.** (E)** Statistical analysis of serum pro-inflammatory and anti-inflammatory factor concentrations in 5×FAD mice treated with Mn_3_O_4_ nanozymes for 4 and 8 weeks by flow cytometry, n = 3 per group. Data are presented as mean ± SEM. Multiple t-tests were used for multigroup comparisons.^ **^p < 0.01 and ^*^p < 0.05, and ns (no significance).

To further investigate the ability of Mn_3_O_4_ nanozymes to continuously modulate microglial phenotypes during 8 weeks of treatment and its potential regulatory mechanisms, we treated 5×FAD mice with Mn_3_O_4_ nanozymes for 4 and 8 weeks according to the scheme in **Figure 5A**. To evaluate the sustained regulatory effect of Mn_3_O_4_ nanozymes on microglial phenotypes, we detected changes in microglial phenotypes in the hippocampus and prefrontal cortex by immunofluorescence. Immunofluorescence results showed that after 4 weeks of treatment, Mn_3_O_4_ nanozymes had no significant effect on the positive area of M1 microglia (**Figure S22**), but significantly increased the positive area of M2 phenotype microglia in the hippocampus and prefrontal cortex (*p* < 0.01, **Figure 5B-D**). However, in the hippocampus and prefrontal cortex of 5×FAD mice, the positive area of M2 phenotype and M1 phenotype microglia did not increase significantly after 8 weeks of Mn_3_O_4_ nanozymes treatment (**Figure 5B-D** and **Figure S22**). This further demonstrated that Mn_3_O_4_ nanozymes can modulate microglial phenotypes well during the 4-week treatment, but this modulation is not sustained.

Under the pathological state of Aβ, microglia activate inflammatory pathways and release large amounts of pro-inflammatory factors, thereby promoting the polarization of microglia toward the pro-inflammatory M1 phenotype 49. Therefore, the regulation of microglial phenotype by Mn_3_O_4_ nanozymes may be related to its inhibition of pro-inflammatory factors. We next quantified the levels of pro-inflammatory cytokines in the serum of 5×FAD mice after 4 and 8 weeks of treatment with Mn_3_O_4_ nanozymes. The results showed that serum interferon-gamma (IFN-γ) and interleukin-17A (IL-17A) levels were significantly reduced after 4 weeks of Mn_3_O_4_ nanozyme treatment (*p* < 0.05). In addition, Mn_3_O_4_ nanozymes did not show suppression of inflammatory responses after 8 weeks of treatment (**Figure 5E, Figure S23**). Therefore, Mn_3_O_4_ nanozymes promote the transition of microglia to the anti-inflammatory M2 phenotype at 4 weeks of treatment by inhibiting the release of pro-inflammatory factors.

### Mn_3_O_4_ nanozymes inhibited inflammatory responses by suppressing ROS generation via the TLR4/NOX2 pathway

**Figure 6:**
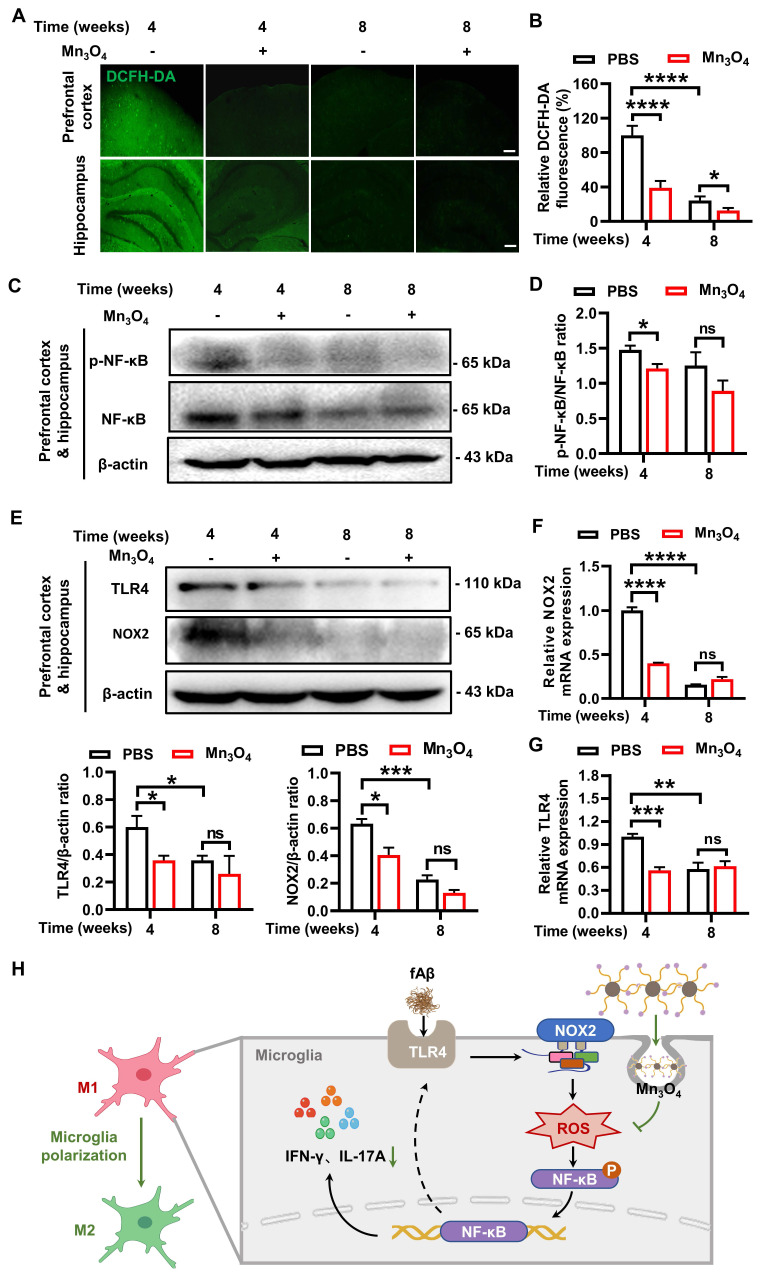
** Mn_3_O_4_ nanozymes regulated microglial phenotype by inhibiting the TLR4/NOX2 pathway, thereby reducing ROS generation and NF-κB-mediated inflammatory responses. (A-B)** Representative images and statistical analysis of fluorescence imaging of prefrontal cortex and hippocampus slices incubated with DCFH-DA probe in 5×FAD mice treated with Mn_3_O_4_ nanozyme for 4-8 weeks, n = 6 per group, scale bar = 100 μm. **(C-D)** Immunoblotting results and densitometric analysis of p-NF-κB and NF-κB in the prefrontal cortex and hippocampus of 5×FAD mice treated with Mn_3_O_4_ nanozymes for 4-8 weeks, n = 3 per group. **(E)** Immunoblotting results and densitometric analysis of NOX2 and TLR4 in the prefrontal cortex and hippocampus of 5×FAD mice after 4-8 weeks of Mn_3_O_4_ nanozyme treatment, n = 3 per group. **(F-G)** Relative mRNA expressions of NOX2 and TLR4 in the prefrontal cortex and hippocampus of 5×FAD mice after 4-8 weeks of Mn_3_O_4_ nanozyme treatment, n = 4 per group.** (H)** Schematic diagram of Mn_3_O_4_ nanozymes inhibiting inflammatory response and regulating microglia phenotype through TLR4/NOX2 pathway. Data are presented as mean ± SEM. Multiple t-tests were used for multigroup comparisons. ^****^p < 0.0001, ^***^p < 0.001, ^**^p < 0.01, ^*^p < 0.05, and ns (no significance).

Although Mn_3_O_4_ nanozymes can promote the regulation of microglial phenotype by inhibiting pro-inflammatory factors, the exact molecular mechanism of its anti-inflammatory effect remains to be further studied. As a nanozyme with antioxidant enzyme mimetic activity, Mn_3_O_4_ nanozyme can exert antioxidant effects by reducing intracellular ROS 50. ROS can increase the release of pro-inflammatory factors and promote the differentiation of microglia into M1 type by activating the downstream NF-κB signaling pathway 51. Therefore, the inhibition of pro-inflammatory factors by Mn_3_O_4_ nanozymes may be intrinsically linked to the suppression of cellular ROS. To validate this hypothesis, we quantitatively measured the ROS levels in the prefrontal cortex and hippocampus of 5×FAD mice. The results of DCFH-DA fluorescence staining revealed that the ROS level in the brain tissue of mice treated with Mn_3_O_4_ nanozymes for 4 and 8 weeks was significantly reduced (*p* < 0.0001, *p* < 0.05, **Figure 6A-B**). We further examined the expression levels of phosphorylated NF-κB p65 (p-NF-κB) and NF-κB in the prefrontal cortex and hippocampus of 5×FAD mice after 4 and 8 weeks of Mn_3_O_4_ nanozyme treatment. The results showed that Mn_3_O_4_ nanozymes significantly reduced the ratio of p-NF-κB to NF-κB after 4 weeks of treatment (*p* < 0.05, **Figure 6C-D, Figure S24A-B**). These results suggest that the Mn_3_O_4_ nanozymes can reduce ROS levels in the brain within the first 4 weeks of treatment and further inhibit the production of pro-inflammatory factors by suppressing the expression of NF-κB.

Although the results showed that Mn_3_O_4_ nanozymes can reduce intracellular ROS levels, the mechanism of ROS reduction remains to be elucidated. Studies have shown that ROS overproduction is mainly mediated by the activation of NOX2 subunits, and Nrf2 regulates NOX2-mediated ROS overproduction 52. To explore the mechanism of Mn_3_O_4_ nanozymes reducing ROS production, we detected the protein and mRNA expression levels of NOX2 and Nrf2 in the prefrontal cortex and hippocampus of 5×FAD mice. The results showed that after 4 weeks of Mn_3_O_4_ nanozyme treatment, the mRNA (*p* < 0.0001, **Figure 6F**) and protein expression levels (*p* < 0.05, **Figure 6E** and **S24D**) of NOX2 in the brain tissue of 5×FAD mice were significantly reduced. However, no significant changes were observed in the mRNA and protein expression levels of Nrf2 (**Figure S25**). Therefore, the inhibition of ROS generation by Mn_3_O_4_ nanozymes mainly involves the suppression of NOX2 expression, and has no obvious correlation with the regulation of Nrf2.

Studies have shown that TLR4 recognition of fAβ can induce microglial polarization and exacerbate AD pathology by activating downstream inflammatory signals 14. NOX2 subunit can also mediate inflammatory responses via ROS 53. Therefore, TLR4 receptors may be an important factor affecting the production of ROS by NOX2 subunits and promoting inflammatory responses. Since Mn_3_O_4_ nanozymes can reduce the expression of NOX2 subunits and ROS levels in the brain of 5×FAD mice, we further explored the effect of Mn_3_O_4_ nanozymes on TLR4 protein expression in 5×FAD mice. The results showed that after 4 weeks of Mn_3_O_4_ nanozyme treatment, the expression of TLR4 in the prefrontal cortex and hippocampus of 5×FAD mice was significantly reduced (*p* < 0.05, **Figure 6E** and **S24C**). This indicates that the inhibitory effect of Mn_3_O_4_ nanozyme on TLR4 receptor is closely related to its inhibitory effect on NOX2 subunit. In fact, ROS can also promote the transcription of TLR4 receptor and its signal transduction by activating the downstream NF-κB signaling pathway 54, 55. Therefore, we further detected the changes in TLR4 mRNA expression in the prefrontal cortex and hippocampus of 5×FAD mice. The results showed that Mn_3_O_4_ nanozyme treatment significantly reduced TLR4 mRNA expression after 4 weeks (*p* < 0.001, **Figure 6G**). In addition, Mn_3_O_4_ nanozymes reduced ROS and inhibited NF-κB activation, thereby inhibiting TLR4 receptor expression, forming a negative feedback to maintain anti-inflammatory effects. Taken together, these findings suggest that Mn_3_O_4_ nanozymes reduce ROS generation and inhibit NF-κB-mediated inflammatory response by inhibiting the TLR4/NOX2 pathway, ultimately regulating microglial phenotypes (**Figure 6H**).

## Discussion

Neuroinflammation is considered to be a crucial factor in fAβ degeneration and AD progression, and controlling the process and timing of neuroinflammation may be key to the success of anti-inflammatory treatment of AD. In this study, we designed a water-soluble ROS scavenger Mn_3_O_4_ nanozyme and demonstrated that the nanozymes promoted the transition of microglial phenotype from M1 to M2 by scavenging ROS. In the nasal treatment experiment of AD transgenic mice, Mn_3_O_4_ nanozymes promoted the polarization of microglia to M2 phenotype after 4 weeks of treatment, while fAβ pathology and cognitive deficits of mice were alleviated after 8 weeks of treatment. This suggests that anti-inflammatory treatment of AD should be carried out in the early stage when the microglial phenotype can be reversed, and longer treatment may achieve the effect of inhibiting AD pathology. In addition, by inhibiting the TLR4/NOX2 pathway to scavage ROS, the M1/M2 phenotype of microglia was regulated. Our study provides molecular insights into the regulation of microglial phenotypic transition by nanozymes, thus opening up new ideas for anti-inflammatory treatment of AD.

Microglial phenotypic transition involves multiple signaling pathways, including TLR4/NF-κB pathway, Nod-like receptor (NLR) pathway, and Janus kinase (JAK)/signal transducer and activator of transcription (STAT) pathway 56. These signaling pathways involved in regulating microglial phenotypic transition can serve as potential targets for regulating neuroinflammation, and blockers for these signaling pathways can serve as regulators 57-59. Compared with traditional anti-inflammatory small molecule compounds, nanomaterials have better bioavailability, targeting, and ability to penetrate the blood-brain barrier (BBB) due to the tunability of their size, physicochemical properties, and surface reactivity 60. Among them, manganese-based nanozymes are not only widely used in drug delivery due to their good stability 61, but can also modify targeted antibodies, polymers, or cell membrane proteins for the diagnosis and treatment of AD 26, 62-64. Due to the lack of ability to penetrate the BBB, manganese-based nanozymes are difficult to directly enter the brain to exert their therapeutic effects 35. Considering that Mn_3_O_4_ nanozymes cannot cross the BBB, we chose to deliver Mn_3_O_4_ nanozymes to the brain via intranasal administration (**Figure S8**), thereby achieving the purpose of inhibiting neuroinflammation and regulating microglial phenotype.

Previous studies have shown that compared with systemic administration, intranasal administration of nanomedicines can effectively improve brain targeting efficiency, and is beneficial for the treatment of various neuropsychiatric diseases 65, 66. This is mainly because the drug is directly transported from the nasal cavity to the brain through the olfactory and trigeminal nerve pathways, thereby effectively improving bioavailability 67. For intranasal administration, although sufficient blood supply to the nasal mucosa is conducive to drug absorption, the amount of drug acceptable in the nasal cavity is small, so it is necessary to increase the drug concentration during nasal administration 68. For poorly soluble drugs, the polarity of drugs can be increased by modifying the surfactant groups, thereby improving the solubility of drugs 69. In addition, modifying the surfactant groups of drugs is also beneficial to improve the brain entry efficiency and achieve better therapeutic effects 70, 71. Considering that Mn_3_O_4_ is insoluble in water, we used DSPE-PEG-Mal for surface modification to increase the water solubility of Mn_3_O_4_ nanozymes (**Figure 1A and Figure S1D**). Brain targeted delivery of Mn_3_O_4_ nanozymes was achieved through nasal administration (**Figure S9**).

In LPS-induced neuroinflammation, the key receptor TLR4 on the cell membrane can mediate inflammatory responses, thereby promoting the polarization of microglia to the M1 phenotype 72. In this study, we found that after LPS injection, the expression level of TLR4 and the number of M1 microglia in the hippocampal brain tissue of C57BL/6J mice increased significantly, and Mn_3_O_4_ nanozyme treatment regulated microglial phenotype of the hippocampal tissue of LPS inflammation model mice by inhibiting TLR4 expression levels (**Figure 2B-F**). However, the mechanism of Mn_3_O_4_ nanozymes regulating microglial phenotypes by the inhibition of TLR4 is still unclear. Studies have shown that Aβ protein can act as a ligand to activate microglial TLR4 receptor, thereby promoting inflammatory responses 49. At the same time, ROS produced by NOX2 is also believed to mediate inflammatory responses 53. Therefore, we speculated that activation of the TLR4 receptor may further induce NOX2 to produce a large amount of ROS, thereby exacerbating the inflammatory response. We evaluated the expression of TLR4 in the brain tissue of 5×FAD transgenic mice and found that TLR4 expression was significantly upregulated compared with wild-type mice (**Figure 3A**). Interestingly, the expression level of NOX2 was also significantly upregulated (**Figure S26**), indicating that Aβ further promoted the expression of NOX2 after binding to the TLR4 receptor. After 4 weeks of treatment of 5×FAD mice, we confirmed that Mn_3_O_4_ nanozymes inhibited the transcription and expression of TLR4 and further decreased ROS generation by reducing the expression of NOX2 subunits (**Figure 6A-B and Figure 6E-G**). Since ROS can aggravate the inflammatory response by activating downstream NF-κB, it can affect the polarization of microglia 13, 73. Our results further showed that Mn_3_O_4_ nanozymes inhibited the downstream NF-κB-mediated inflammatory response (**Figure 5E and 6C-D**) and promoted the polarization of microglia to the M2 phenotype (**Figure 5B-D**). Therefore, Mn_3_O_4_ nanozymes reduce the level of ROS produced by NOX2 subunits by inhibiting the transcription and expression of TLR4 receptors, thereby alleviating the downstream NF-κB-mediated inflammatory response and ultimately promoting the polarization of M2 microglia.

Intranasally administered Mn_3_O_4_ nanozymes rapidly inhibited the overproduction of ROS and inflammatory cytokines after 4 weeks of treatment and promoted the polarization of microglia toward the M2 phenotype. However, after 8 weeks of continuous treatment, Mn_3_O_4_ nanozymes did not show a sustained inhibitory effect as expected (**Figure 5B-D**). Although Mn_3_O_4_ nanozymes we synthesised did not specifically target microglia (**Figure S10**), after intranasal administration for 36 h, the Mn_3_O_4_ nanozyme was almost completely excluded from the body (**Figure S9**), and no accumulation of manganese ions was detected in the brain (**Figure S27**). This suggests that the Mn_3_O_4_ nanozyme will not accumulate in other peripheral organs or be metabolized to manganese ions to activate immune pathways, thereby affecting its regulatory effects on the microglial phenotypes. In the early stage of AD, microglia have well-trained immune function to external stimuli, which is mainly manifested as microglia activation and enhanced release of pro-inflammatory factors, thereby aggravating the pathological process. In the late stage of AD, continuous stimulation of high concentrations of pathological proteins causes microglia to transform into an immune tolerance phenotype, resulting in desensitization and ultimately leading to a reduction in inflammatory factors 74-76. Therefore, the reduced efficacy of Mn_3_O_4_ nanozyme treatment after 8 weeks may be related to the innate immune memory of microglia. Regarding the signaling pathways related to innate immune memory, studies have shown that when the TLR4 receptor is repeatedly stimulated by ligands, inflammatory factors are sharply reduced, thereby weakening the antigen presentation ability of cells, that is, in a state of cellular immunesuppression 77, 78. In our study, we detected a significant decrease in TLR4 receptor expression (**Figure 6E**) and pro-inflammatory cytokine secretion (**Figure 5E**) with the development of AD, suggesting that developed immune tolerance after 8 weeks of treatment. Due to the immune tolerance of TLR4 receptor signaling, its downstream NOX2 subunit could not form a signalling complex (**Figure 6E-F**), thereby reducing the production of ROS (**Figure 6A-B**). At the same time, the reduced ROS further enhanced the immune tolerance of microglia by inhibiting the transcription of TLR4 receptors in the NF-κB pathway (**Figure 6G**). Therefore, the anti-inflammatory effect could not be sustained during the 8-week treatment with Mn_3_O_4_ nanozyme. Regarding immune tolerance mediated by reduced TLR4 receptor sensitivity, studies have found that immune responses can be restored by immunostimulatory therapy 79. For example, the use of hydroxyapatite nanoparticles can eliminate the endotoxin tolerance of macrophages and enhance the body's response to LPS stimulation by increasing TLR4 receptor signaling 80. Furthermore, the increase of miR-146a can induce microglia to develop tolerance to Aβ/LPS and weaken the inflammatory response, while the down-regulation of miR-146a can restore the body's sensitivity to inflammatory response 81. Therefore, although the anti-inflammatory effect of Mn_3_O_4_ nanozymes weakened after 8 weeks of treatment, if used in combination with immunostimulatory therapies, Mn_3_O_4_ nanozymes may be able to unlock the immune tolerance of microglia and restore their anti-inflammatory treatment of AD. This aspect deserves further exploration in the future.

Given that Aβ deposition triggers a series of neuroinflammatory responses in the brain mediated by activated microglia 82, Aβ plaque load can also be reduced by regulating microglial phenotype 83. In this study, we further investigated the changes in fAβ plaques in 5×FAD mice after Mn_3_O_4_ nanozyme treatment. The results showed that Mn_3_O_4_ nanozymes first exhibited a modulatory effect on microglial phenotype and could not effectively reduce fAβ load in 4 weeks of treatment (**Figure 3D-F**). However, after 8 weeks of treatment, Mn_3_O_4_ nanozymes significantly reduced fAβ plaques and improved learning memory functions (**Figure 4**). The above results suggest that Mn_3_O_4_ nanozymes may reduce fAβ aggregation by regulating the phenotype of microglia. Given that we have demonstrated that the anti-inflammatory effect of Mn_3_O_4_ nanozymes is mainly exerted by inhibiting TLR4 receptors and promoting the transformation of microglia to M2 phenotype (**Figure 5B-D, Figure 6E-G**). However, the effect of TLR4 receptors on Aβ production on the cell membrane surface is indirectly produced by upregulating the expression of β-secretase 1 (BACE1) 84. Therefore, the inhibitory effect of Mn_3_O_4_ nanozymes on TLR4 receptor cannot directly eliminate fAβ, but indirectly reduces the production of Aβ in the brain by inhibiting the expression of BACE1, which also leads to the improvement of fAβ pathology in mice after 8 weeks of treatment. In the future, anti-inflammatory therapy strategies combined with antibodies directly targeting Aβ may help to effectively alleviate AD pathology and inflammatory responses.

In conclusion, our study found that Mn_3_O_4_ nanozymes can regulate the microglial phenotypes and reduce neuroinflammation in 5×FAD mice through anti-inflammatory treatment within 4 weeks. Although the regulation effect of Mn_3_O_4_ nanozymes on microglial phenotype in this study weakened after 8 weeks of treatment, it significantly reduced fAβ pathology in 5×FAD mice and ameliorated learning and memory impairment, providing a new insight for anti-neuroinflammatory treatment of AD. In addition, our study found that the regulation of microglial phenotype and reduction of neuroinflammation by Mn_3_O_4_ nanozymes was mainly achieved by inhibiting the TLR4/NOX2 pathway for ROS clearance. This inhibition reveals the molecular mechanism of antioxidant nanomaterials regulating microglial phenotype and provides an experimental basis for the in-depth exploration of the relationship between neuroinflammation and AD pathology.

## Supplementary Material

Supplementary methods and figures.

## Abbreviations

AD: Alzheimer's disease
Aβ: amyloid-β
fAβ: fibrillar β-amyloid protein
CAT: catalase
GSH-Px: glutathione peroxidase
WT: wild-type
IFNγ: interferon gamma
LPS: lipopolysaccharide
NO: nitric oxide
ROS: reactive oxygen species
NAPDH: nicotinamide adenine dinucleotide phosphate
NOX2: NADPH oxidase 2
TLR4: toll-like receptor 4
SOD: superoxide dismutase
DCFH-DA: dichlorofluorescin diacetate
IMDM: Iscove's Modified Dubecco's Medium
FBS: fetal bovine serum
ECL: enhanced chemiluminescence liquid
PBS: phosphate buffer saline
DAPI: 4′,6-diamidino-2-phenylindole
TEM: transmission electron microscope
XRD: X-ray diffraction
XPS: X-ray photoelectron spectroscopy
DAB: 3,3-diaminobenzidine
CBA: cytometric bead array
MWM: morris water maze
RT-qPCR: quantitative real-time polymerase chain reaction
DLS: dynamic light scattering
NF-κB: nuclear factor kappa-B
IL-17A: interleukin-17A
Nrf2: nuclear factor erythroid 2-related factor 2
BACE1: beta-secretase 1
M1: classically activated
M2: alternatively activated
RIPA: radio immunoprecipitation assay
NLR: nod-like receptor
JAK: janus kinase
STAT: signal transducer and activator of transcription
BBB: blood-brain barrier
Mn(Ac)_2_: manganese acetate
OA: oleic acid
BSA: bovine serum albumin
ALT: alanine aminotransferase
AST: aspartate aminotransferase
BUN: blood urea nitrogen
CREA: creatinine
·O_2_^-^: superoxide anions
·OH: hydroxyl radicals
IHC: immunohistochemistry
IF: immunofluorescence
WB: western blotting
IN: intranasal
H&E: hematoxylin and eosin
DSPE-PEG-Mal: 1,2-distearoyl-sn-glycero-3-phosphoethanolamine-N-[Maleimide(polyethylene-glycol)-2000]
DSPE-PEG-NH2: 1,2-distearoyl-sn-glycero-3-phosphoethanolamine-N-[amino(polyethylene-glycol)-2000]
ICP-OES: inductively coupled plasma-optical emission spectroscopy
