# Supplementary material for: Mn3O4 nanozyme-based anti-inflammatory therapy modulates microglial phenotype by downregulating TLR4/NOX2 expression and further alleviates Alzheimer's disease pathology
Source: Theranostics. 2025 Jun 20;15(15):7467–88. doi: 10.7150/thno.112213 (PMC12315813; doi:10.7150/thno.112213)
Supplement: Supplementary file 1 — Supplementary methods and figures. [file thnov15p7467s1.pdf]

Supporting Information

Mn_3O_4 nanozyme-based anti-inflammatory therapy modulates microglial phenotype by downregulating TLR4/NOX2 expression and further alleviates Alzheimer's disease pathology

Jun Xie[1#], Kai Cao[1#], Luman Liu[1], Liding Zhang[2], Ying Yang[3], Hui Gong[1,4], Haiming Luo[1,2,4*]

[1]MOE Key Laboratory for Biomedical Photonics, Wuhan National Laboratory for Optoelectronics, Huazhong University of Science and Technology, Wuhan 430074, China.

[2]State Key Laboratory of Digital Medical Engineering, Key Laboratory of Biomedical Engineering of Hainan Province, School of Biomedical Engineering, Hainan University, Haikou 570228, China.

[3]Department of Pathophysiology, School of Basic Medicine, Key Laboratory of Education Ministry of China/Hubei Province for Neurological Disorders, Tongji Medical College, Huazhong University of Science and Technology, Wuhan 430074, China

[4]HUST-Suzhou Institute for Brainsmatics, JITRI, Suzhou 215123, China.

[#]These authors contributed equally to this work.

[*]Corresponding authors: Haiming Luo; E-mail: hemluo@hust.edu.cn

MOE Key Laboratory for Biomedical Photonics, Wuhan National Laboratory for Optoelectronics, Huazhong University of Science and Technology, Wuhan 430074, China;

This supporting Information includes:

Materials and Methods

Supplementary Figures S1-S27

Materials and Methods

Evaluation of multienzyme-like activity

The SOD-like activity and GSH-Px-like activity of Mn_3O_4 nanozymes were determined by total superoxide dismutase assay kit (#G0104W, Grace Biotechnology, China) and glutathione peroxidase assay kit (#G0204W48, Grace Biotechnology, China) according to the manufacturer's instructions. Finally, the SOD-like activity and GSH-Px-like activity of the nanozyme were calculated by colorimetric method.

The CAT-like activity of Mn_3O_4 nanozymes was assayed by catalase assay kit (#BC0200, Solarbio, China) according to the manufacturer's instructions.

Free radical ($\cdot O_2^-$ and $\cdot OH$) scavenging assay

The total superoxide dismutase detection kit (#G0104W, Grace Biotechnology, China) and the hydroxyl radical scavenging ability detection kit (#BC1320, Solarbio, China) were used to detect the $\cdot O_2^-$ and $\cdot OH$ scavenging rates of Mn_3O_4 nanozymes, respectively, according to the methods provided by the manufacturers.

Stability assessment

Mn_3O_4 nanozymes were incubated continuously at 37 °C for 28 days, and the hydrodynamic diameter, zeta potential, SOD-like activity, and superoxide anion scavenging ability of the nanozymes were measured at the designated time points.

Measurement of ion release

Mn_3O_4 nanozymes (1 mg/mL) were added to a dialysis bag containing simulated nasal electrolyte solution and dialysed in 1 L of deionised water. The dialysate (10 mL) was collected at 1, 3, 7, 14, and 28 days, and the Mn ion concentration was determined by inductively coupled plasma optical emission spectroscopy (ICP-OES, Leeman Laboratory, USA).

Hemolysis test

Mouse blood samples were centrifuged (2,000 rpm, 5 min) to retain blood cells. Then, 0.5 mL of red blood cells were mixed with different concentrations of Mn_3O_4 nanozymes and incubated at room temperature for 3 h. Red blood cells were incubated with PBS and deionised water as negative and positive controls, respectively. The mixture was centrifuged (2,000 rpm, 5 min) and the absorbance of the supernatant was measured at 540 nm using an Epoch microplate spectrophotometer (BioTek, USA). Finally, the hemolysis rate was calculated according to the following formula:

$$Hemolysis\ rate(\%) = \frac{A_{sample} - A_{nagetive}}{A_{positive} - A_{nagetive}} \times 100\%$$

Assessment of manganese ion metabolism

8-week-old C57BL/6J mice were randomly divided into three groups (three mice per group) and each mouse was intranasally administered with 60 µL of Mn_3O_4 nanozymes (1 mg/mL). Mouse brain tissues were collected at 3, 6, and 36 h after nasal administration. After weighing, 1 mL of RIPA was added to every 100 mg of brain tissue for homogenization. The supernatant was collected by centrifugation (12, 000 rpm, 10 min) and dialyzed with 100 mL of deionised water for 24 h. The Mn ion concentration was examined by ICP-OES (Leeman Labs, USA).

Cy3 labeling of Mn_3O_4 nanozymes

Cy3 labeling of Mn_3O_4 nanozymes was achieved by the reaction of Cy3 with aminated Mn_3O_4. Briefly, 1 mL of Mn_3O_4-cyclohexane was mixed with an equal volume of ethanol and centrifuged at 10,000 rpm for 10 min. The pellet was dissolved in 1 mL of chloroform and mixed with 25 mg of DSPE-PEG-NH_2 dissolved in 2 mL of chloroform. The solution was stirred at 150 rpm for 1 h and the residual chloroform was removed using a nitrogen blower. The dried sample was ultrasonically dispersed with 5 mL of PBS and centrifuged at 10,000 rpm for 10 min to obtain the aminated Mn_3O_4 nanozymes. The pH value of 1 mL of aminated Mn_3O_4 nanozymes was adjusted to 8.5-9.0, and Cy3-N-hydroxysuccinimide dissolved in 10 µL of dimethyl sulfoxide was added to react at room temperature in the dark for 4 h. After 4 h of reaction, the mixture was dialysed with saline using a 1 kDa dialysis bag to obtain the Cy3-labeled Mn_3O_4 nanozymes.

***In vivo* distribution investigation**

Eight-week-old C57BL/6J mice were randomly divided into three groups, with five mice in each group. Each mouse was intranasally administered with 60 µL of Cy3-labeled Mn_3O_4 nanozymes each time. The mice were executed at 3, 6, and 36 h after administration, and the major organs (heart, liver, spleen, lung, kidney, intestine, stomach, and brain) were removed to detect the fluorescence signal in these tissues using the MOIS HT small animal in vivo optical imaging system (RWD Life science, China).

The brain tissue of mice administered intranasally Mn_3O_4 nanozymes for 6 h was fixed with 4% paraformaldehyde, and coronal sections of the hippocampus were prepared. Afterwards, brain tissue sections were incubated with Iba-1 (microglia-specific marker, 1:500) and NeuN (neuron-specific marker, 1:500) antibodies dispersed in 1% BSA at 4 °C overnight. After washing with PBST, the sections were incubated with Alexa Fluor 488-labeled goat anti-rabbit IgG (1:500) for 1 h at room temperature and counterstained with DAPI. Finally, the co-localization of Mn_3O_4 nanozymes with neurons and microglia was observed on a FV3000 confocal microscope (Olympus, Japan).

Mn_3O_4 nanozyme nasal administration regimen

C57BL/6J mice were randomly divided into sham-operated and LPS-injected groups, and 4 μL of saline or LPS was stereotaxically injected into the hippocampus. Mice in the LPS injection group were given 60 μL PBS or Mn_3O_4 nanozymes (1 mg/ml) intranasally as a preventive drug before 1 h of stereotactic injection of LPS in the hippocampus. After stereotactic injection of LPS in the hippocampus, mice were intranasally administered with PBS or Mn_3O_4 nanozymes at the same dose every other day (three times a week). In addition, for mice in the sham-operated group, equal amount of Mn_3O_4 nanozymes were given in the same manner before and after the hippocampal injection of saline. All mice were treated for 7 days. All mice were executed 1 h after the last intranasal administration, and the hippocampal tissue was isolated or cut to evaluate the microglial phenotype and neuronal morphology.

5×FAD mice were treated with 60 μL Mn_3O_4 nanozymes (1 mg/ml) intranasally every other day (three times a week) for 1, 4 and 8 weeks. Control mice were treated with equal amount of PBS under the same conditions. For mice treated for 1 and 4 weeks, mice were executed 1 h after the last intranasal administration, and the hippocampus and prefrontal cortex were sliced to evaluate microglial phenotype and fAβ pathology. For mice treated for 8 weeks, behavioral tests were conducted 1 h after the last intranasal administration, and subsequently the mice were executed and the hippocampus and prefrontal cortex were separated or sectioned to evaluate microglia phenotype and fAβ pathology.

Evaluation of microglial polarization in the brain

To evaluate the effect of Mn_3O_4 nanozymes on the polarization state of microglia, we focused on the changes in the expression of CD86 and CD206 in microglia in the mouse brain before and after treatment. For LPS-induced inflammation model mice or 5×FAD treated with Mn_3O_4 nanozymes, CD86 and CD206 positive cells in the hippocampus were observed by immunohistochemistry (IHC). Subsequently, the expression of CD86 and CD206 proteins in the hippocampus was quantitatively analyzed by western blotting to evaluate the changing trend of microglial phenotype.

Blood biochemical analysis

All mice were anesthetized and eye blood was collected. After 2 h of whole blood collection, the blood was centrifuged at 5,000 rpm for 10 min to collect the upper serum. Serum alanine transaminase (ALT), aspartate transaminase (AST), blood urea nitrogen (BUN), and creatinine (CREA) were detected by automatic biochemical analyzer (Rayto Life and Analytical Sciences, China).

***In vivo* biosafety of Mn_3O_4 nanozymes**

To assess the toxicity of Mn_3O_4 nanozymes in mice, major organs (brain, heart, liver, spleen, lungs, and kidneys) of mice were removed after continuous nasal administration of Mn_3O_4 nanozymes for 24 h or 8 weeks, and the tissues were fixed with 4% paraformaldehyde and subjected to paraffin sections. In addition, to further evaluate the biosafety of Mn_3O_4 nanozymes on nasal epithelial cells. We collected nasal tissues from mice with continuous nasal administration of nanozymes for 8 weeks, fixed with 4% paraformaldehyde overnight and then decalcified and paraffin-embedded in nasal bone. The proximal part of the nasal airway was selected for paraffin section. All paraffin sections were stained with hematoxylin and eosin (G1120, Solarbio, China). Finally, the sections were observed under an optical microscope (Olympus, Japan).

Statistical analysis

All statistical analyses were performed using GraphPad Prism 9.0 software, and the results are presented as mean ± SEM. Differences between two groups were compared using unpaired t-test. For additional data of multigroup comparisons, one-way analysis of variance was performed, followed by Tukey's test to compare the differences. Statistical significance is indicated in the figure with $^{*}p < 0.05$, $^{**}p < 0.01$, $^{***}p < 0.001$, and $^{****}p < 0.0001$, while n.s. indicates no significance.

Supplementary Figures

Supplementary Figure 1

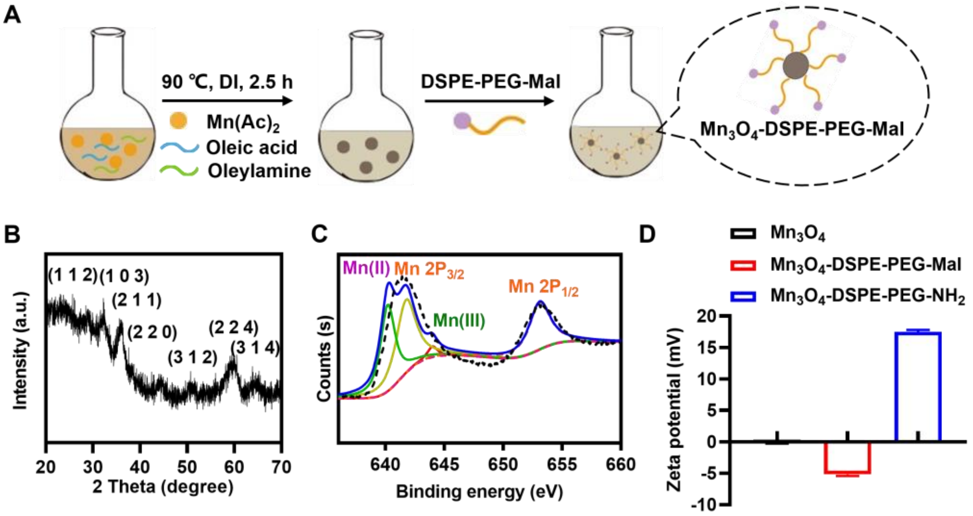

Figure S1. Synthesis and characterization of Mn_3O_4 nanozymes. (A) Schematic diagram of Mn_3O_4 nanozymes. **(B)** The crystal structure of Mn_3O_4 nanozymes corresponded to the crystal planes of hausmannite. **(C)** XPS analysis revealed mixed valance states of Mn^{2+} and Mn^{3+}, as well as the higher presence of Mn^{2+} of Mn_3O_4 nanozymes. **(D)** Zeta potential of Mn_3O_4 nanozymes before and after modification with DSPE-PEG-Mal or DSPE-PEG-NH_2 groups, n = 3. Data are presented as mean ± SEM.

Supplementary Figure 2

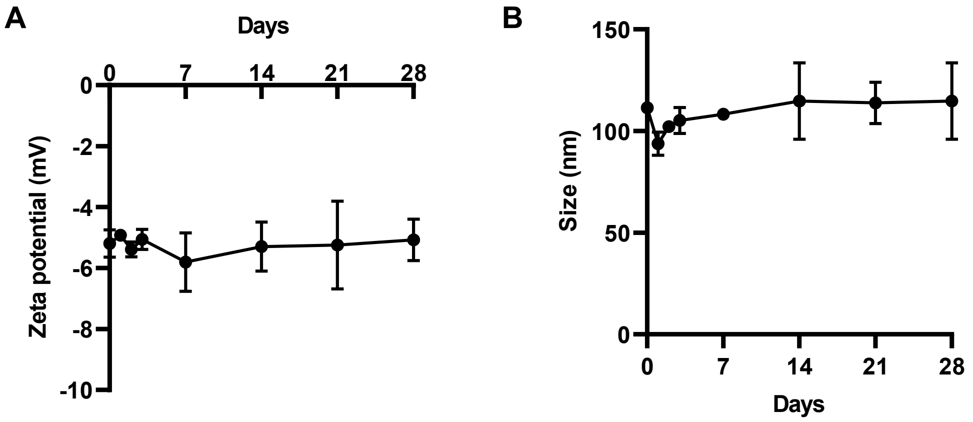

Figure S2. Stability of Mn_3O_4 nanozymes. (A-B) Zeta potential and hydrodynamic diameter of Mn_3O_4 nanozymes during 28 days of incubation at 37 °C, n=3. Data are presented as mean ± SEM.

Supplementary Figure 3

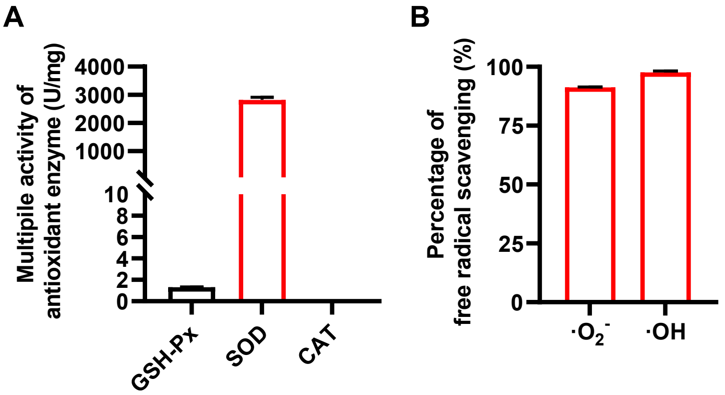

Figure S3. Evaluation of multiple antioxidant enzyme-like activities and multiple free radical scavenging activities of Mn_3O_4 nanozymes. (**A**) The specific enzyme activity test results of SOD-like activity, GSH-Px, and CAT of Mn_3O_4 nanozymes, n = 3. **(B)** Scavenging ability of Mn_3O_4 nanozymes for $\cdot O_2^-$ and ·OH, n = 3. Data are presented as means ± SEM.

Supplementary Figure 4

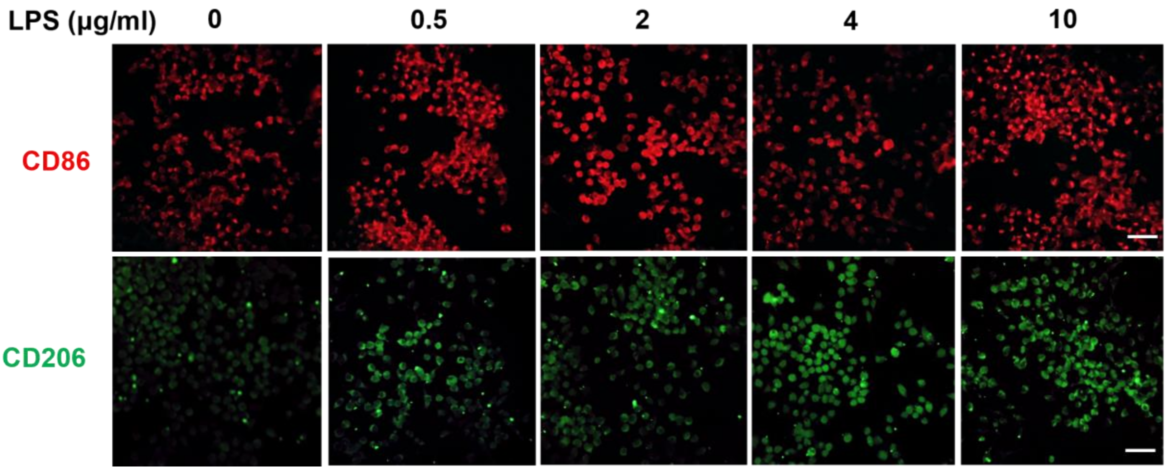

Figure S4. Transformation of M1/M2 phenotype of N9 microglia after LPS stimulation. Representative immunofluorescence images of CD86 and CD206 in N9 microglia after treatment with different concentrations of LPS (0-10 μg/mL) for 24 h, scale bar = 50 μm.

Supplementary Figure 5

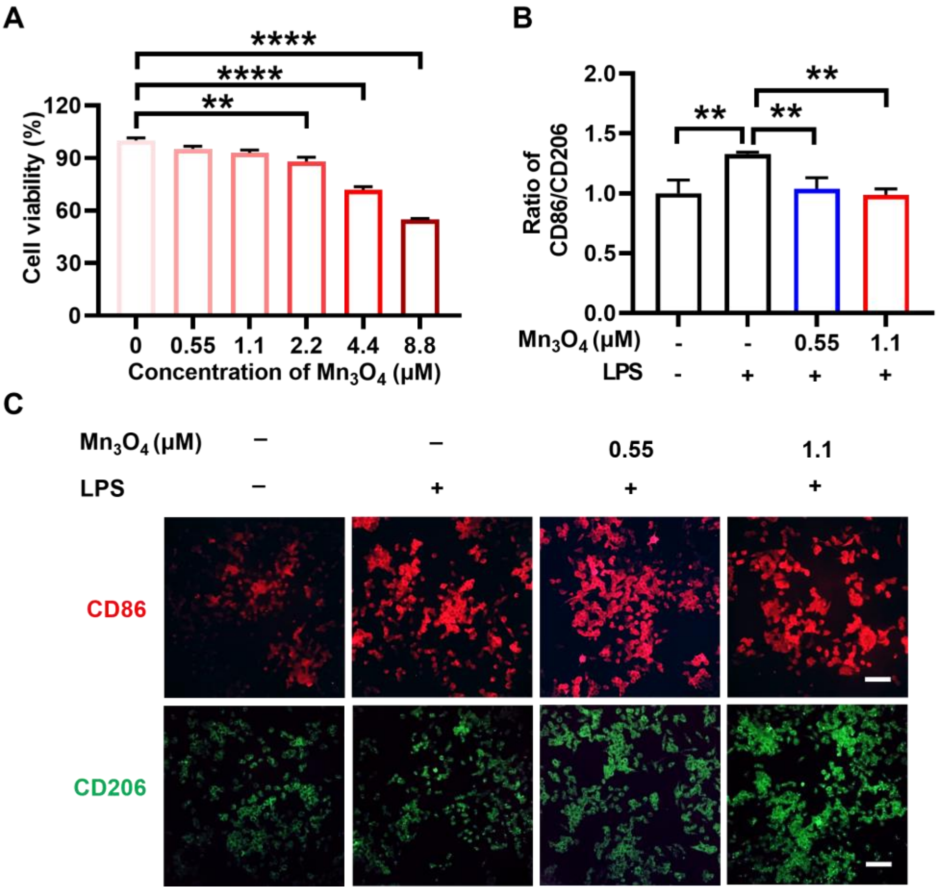

Figure S5. Modulatory effects of Mn_3O_4 nanozymes on the M1/M2 phenotype of LPS-stimulated N9 microglia. (A) Percentage of cell survival after 24 h of treatment of N9 microglia with different concentrations of Mn_3O_4 nanozymes (0.55-8.8 μM). **(B)** Statistical results of CD86/CD206 fluorescence ratio in N9 microglia treated with Mn_3O_4 nanozymes (0.55-1.1 μM) after pre-stimulation with LPS (2 μg/mL). **(C)** Representative immunofluorescence images of M1/M2 phenotype in N9 microglia treated with different concentrations of Mn_3O_4 nanozymes (0.55-1.1 μM) after pre-stimulation with LPS (2 μg/mL), scale bar = 50 μm. Data are presented as mean ± SEM, n = 3. ANOVA was performed for multigroup comparisons. $^{****}P<0.0001$ and $^{**}P<0.01$.

Supplementary Figure 6

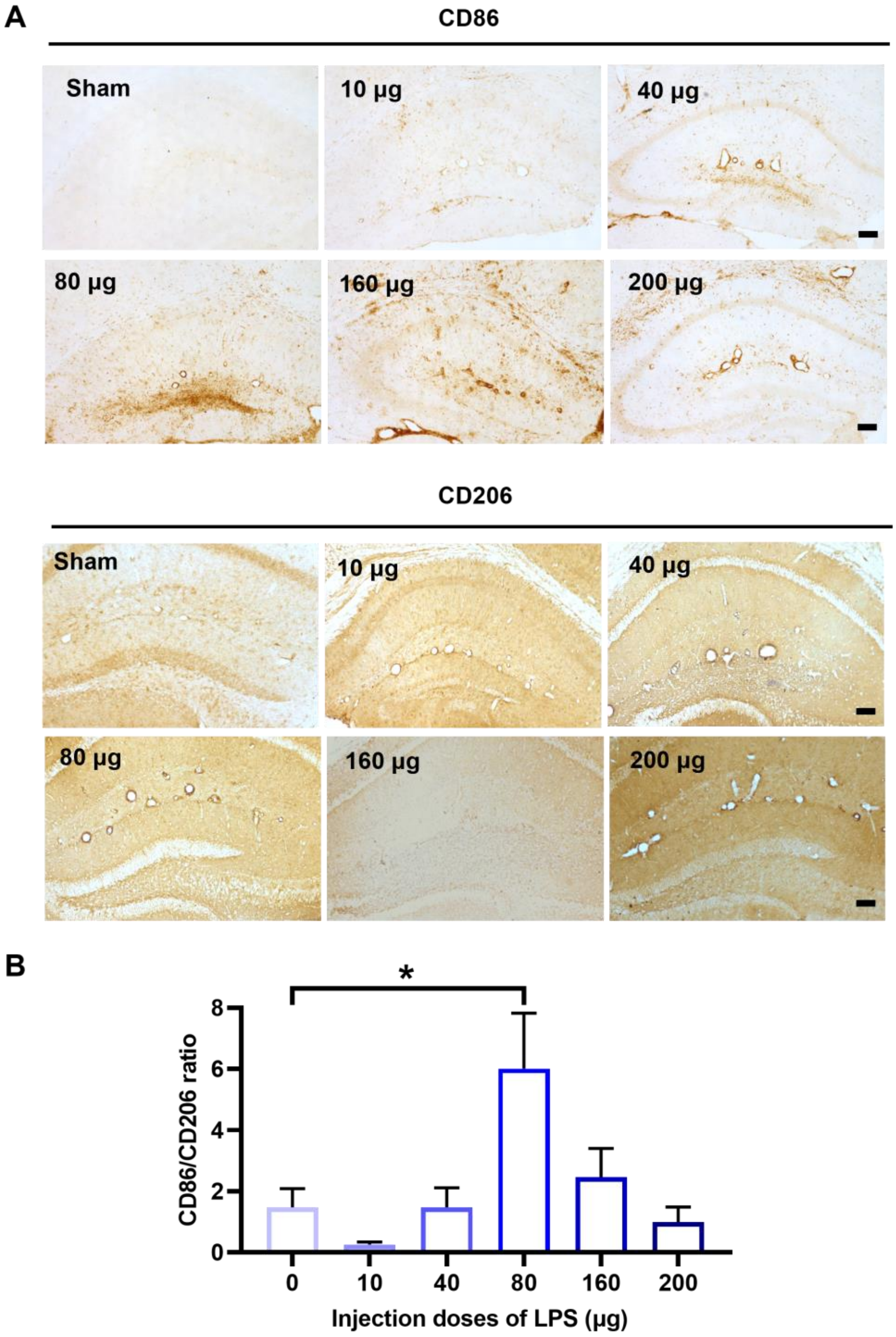

Figure S6. Immunohistochemical staining of hippocampal microglia in C57BL/6J mice after bilateral hippocampal injection of different doses of LPS. (A) Immunohistochemical staining of CD86 and CD206 in hippocampal tissue sections of C57BL/6J mice after bilateral hippocampal injection of different doses of LPS (10-200 μg) for 7 days, scale bar = 100 μm. **(B)** Statistical results of CD86/CD206 immunohistochemical staining. Data are presented as mean ± SEM, n = 3. ANOVA was performed for multigroup comparisons. $^{*}P<0.05$.

Supplementary Figure 7

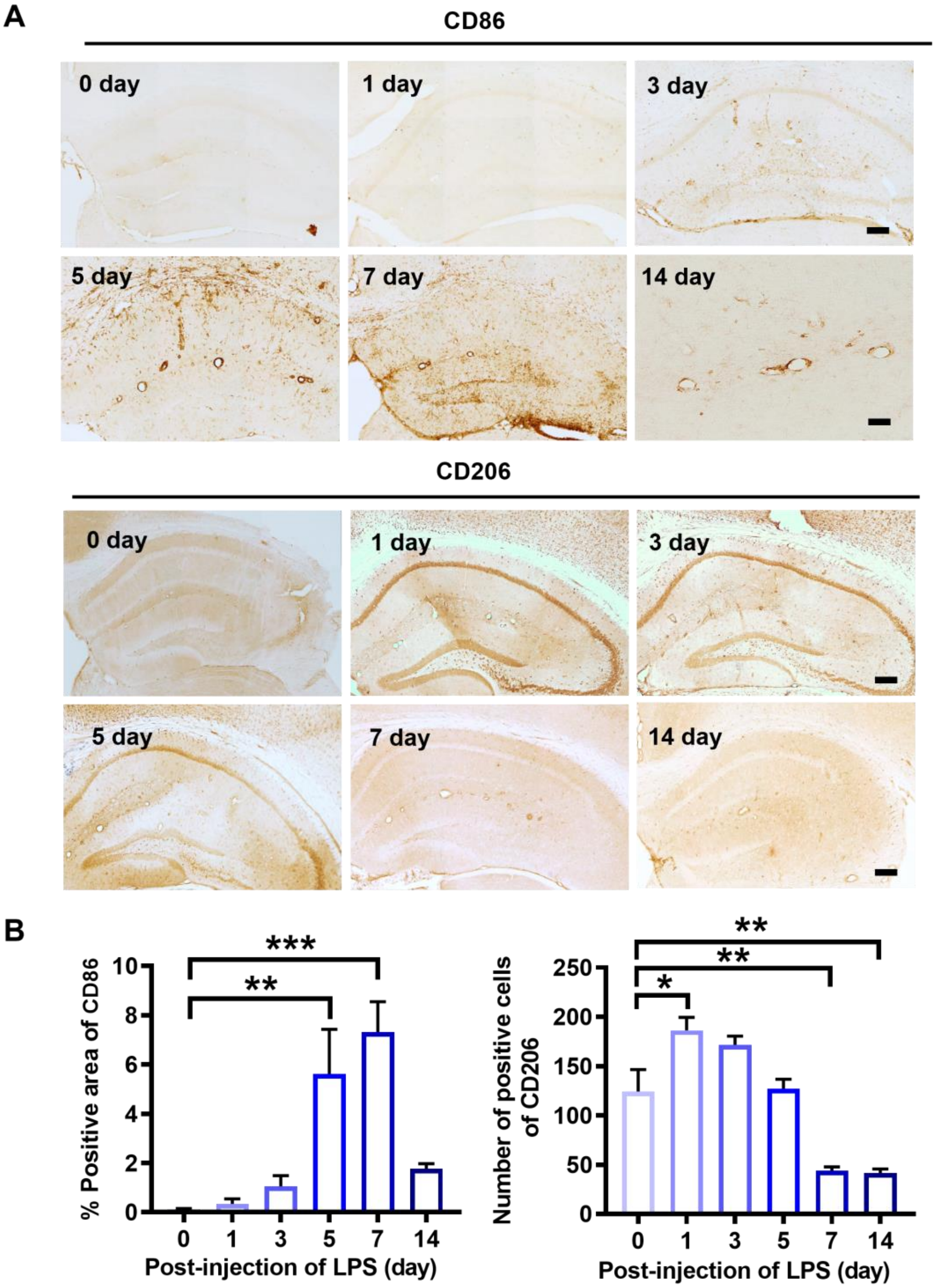

Figure S7. Transformation of microglial M1/M2 phenotype in a time-dependent manner after bilateral hippocampal injection of 80 μg LPS in C57BL/6J mice. (A) Representative immunohistochemical images of CD86 and CD206 on brain sections of mice after bilateral hippocampal injection of 80 μg LPS for 0-14 days, scale bar = 100 μm. **(B)** Statistical data showing the change in CD86 and CD206-positive areas after the above treatments. Data are presented as mean ± SEM, n = 3-4. ANOVA was performed for multigroup comparisons. $^{***}P<0.001$, $^{**}P<0.01$, and $^{*}P<0.05$.

Supplementary Figure 8

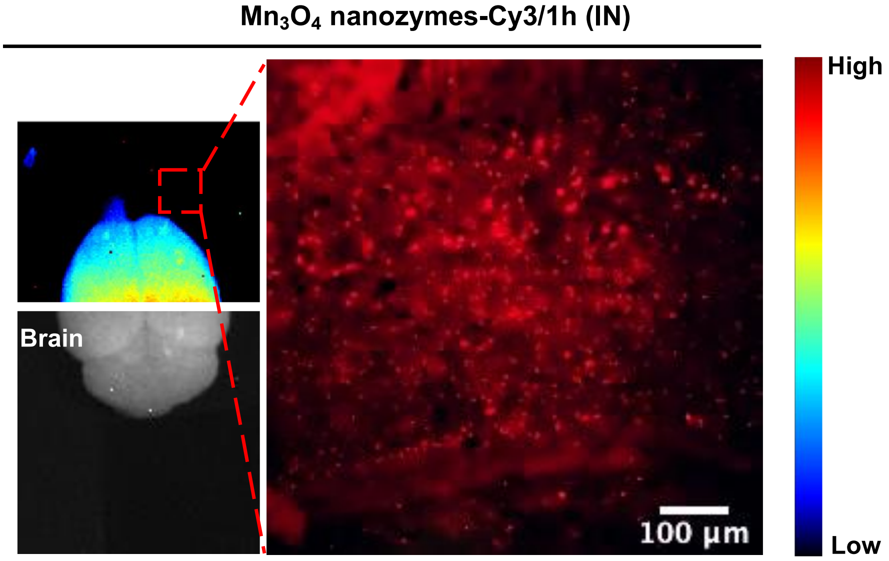

Figure S8. Mn_3O_4 nanozymes could penetrate the blood-brain barrier by intranasal instillation in C57BL/6J mice. Fluorescence imaging of brain (left) and cerebral cortex (right) was performed after 1 h of intranasal injection of Cy3-labeled Mn_3O_4 nanozymes in C57BL/6J mice.

Supplementary Figure 9

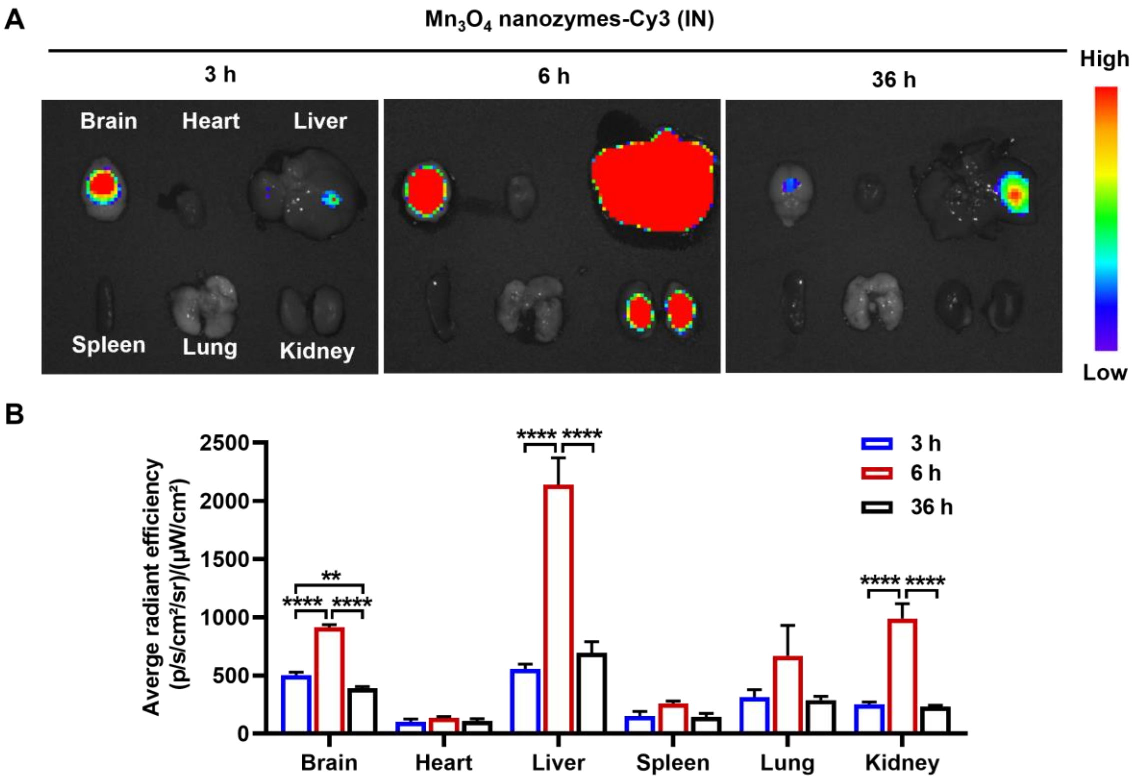

Figure S9. Biodistribution and accumulation of intranasally administered Mn_3O_4 nanozymes *in vivo*. (A) *In vitro* fluorescence imaging of major tissues at different time points

(3, 6, and 36 h) after intranasal administration of Cy3-labeled Mn_3O_4 nanozymes in mice. **(B)** Semi-quantitative analysis of fluorescence intensity of tissue distribution at different time points after intranasal administration of Cy3-labeled Mn_3O_4 nanozymes in mice, n = 5. Data are presented as mean ± SEM. Multiple t-tests were used for multigroup comparisons. $^{****}P<0.0001$ and $^{**}P<0.01$.

Supplementary Figure 10

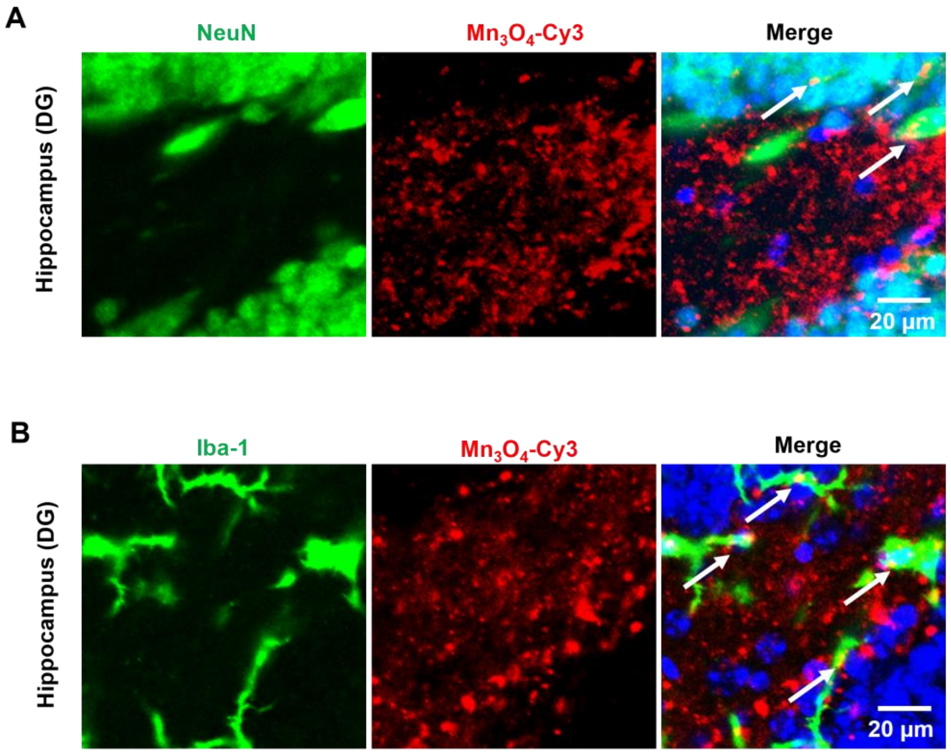

Figure S10. Detection of Cy3-labeled Mn_3O_4 nanozymes in neurons and microglia *in vivo*. (A-B) Representative immunofluorescence images of Cy3-labeled Mn_3O_4 nanozymes (red) entering neurons (stained with NeuN, green) and microglia (stained with Iba-1, green) in the DG region of the hippocampus after 6 h of intranasal administration. Scale bar = 20 μm.

Supplementary Figure 11

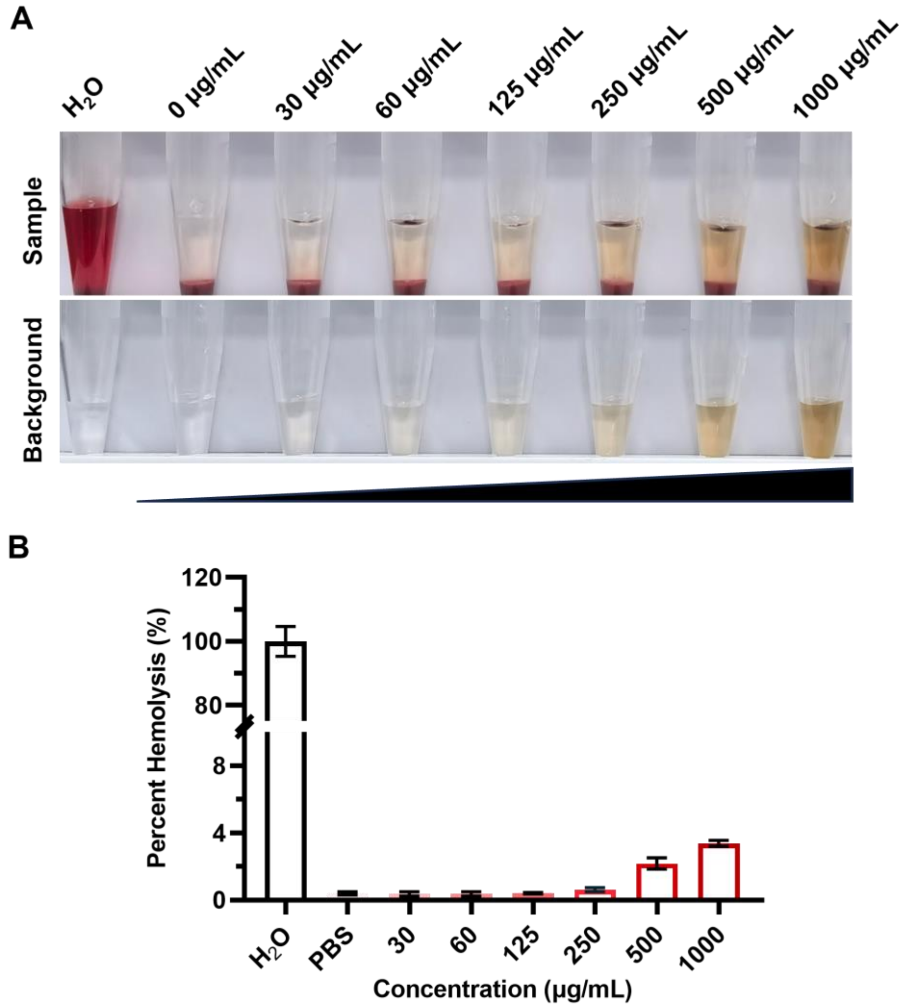

Figure S11. Biocompatibility assay of Mn_3O_4 nanozymes. (A) Photographs of hemolysis test. **(B)** Hemolysis percentage of erythrocyte supernatant after treatment with different concentrations of Mn_3O_4 nanozymes (0-1000 μg/mL), n = 3. Data are presented as mean ± SEM.

Supplementary Figure 12

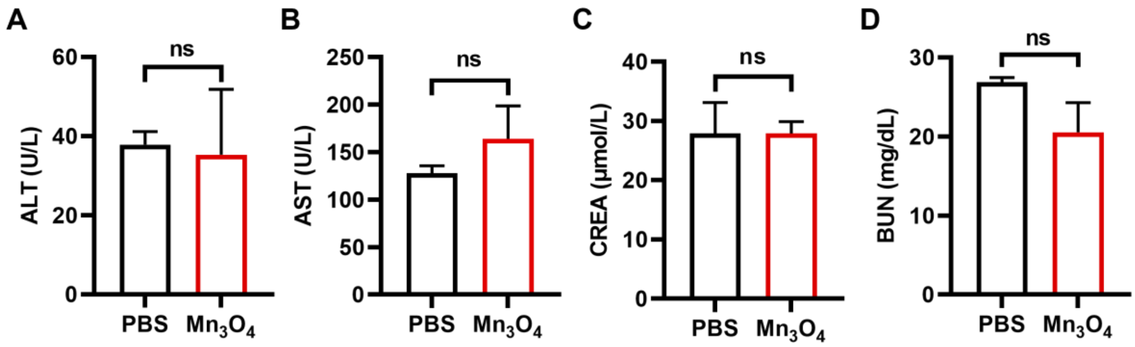

Figure S12. ***In vivo*** **acute hepatorenal toxicity assessment of intranasal administration of Mn_3O_4 nanozymes. (A-D)** Quantitative analysis of liver function (ALT, AST) and renal function (CREA, BUN) in mice after 24 h of intranasal administration of Mn_3O_4 nanozymes. Data are presented as mean ± SEM, n = 3. Unpaired t-test was used for two-group comparisons, ns (no significance).

Supplementary Figure 13

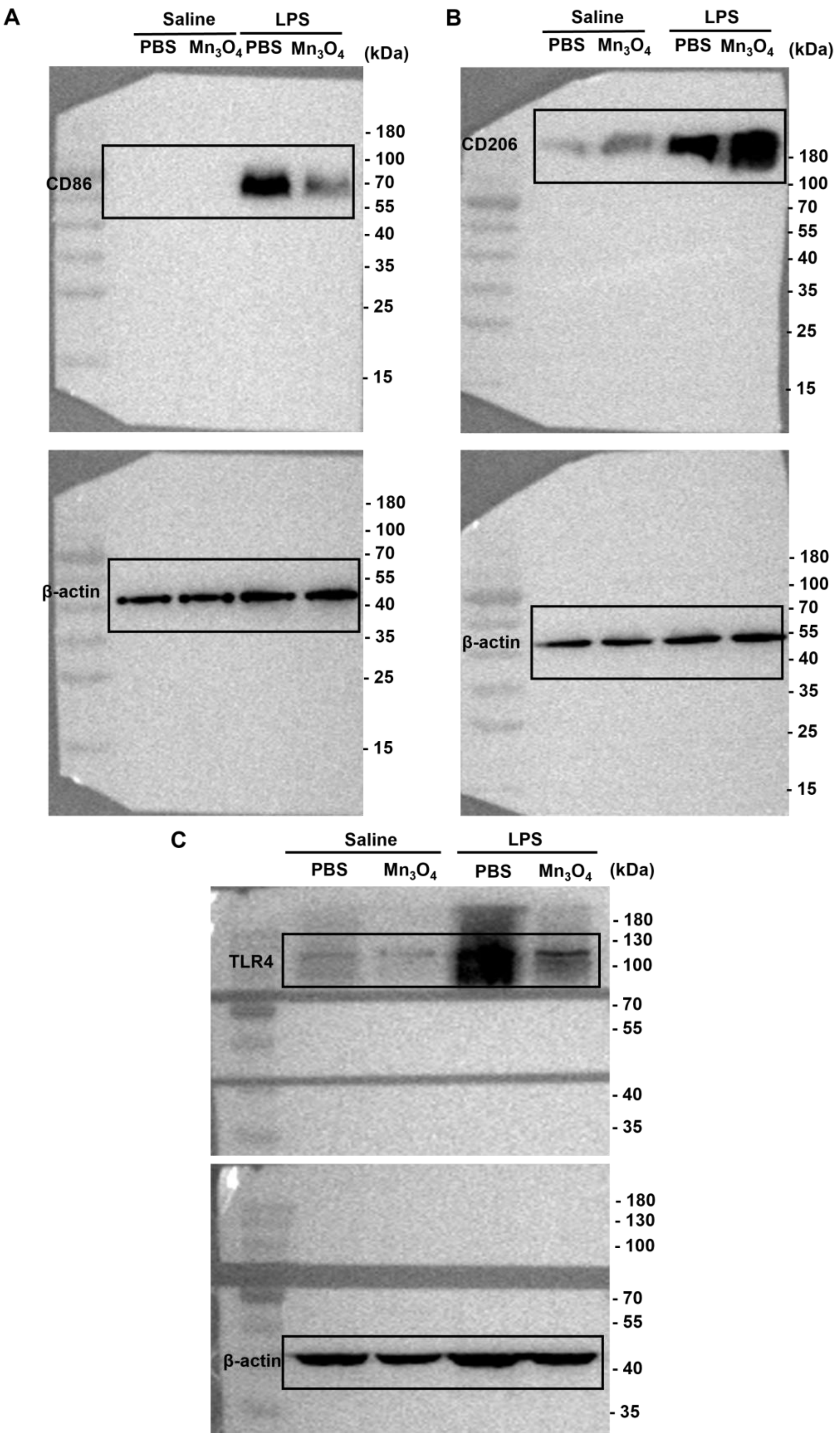

Figure S13. Uncropped western blotting images from Figure 2. Expression of CD86 **(A)**, CD206 **(B),** and TLR4 **(C)** in the hippocampus of LPS-injected mice after treatment with Mn_3O_4 nanozymes in Figure 2C and E.

Supplementary Figure 14

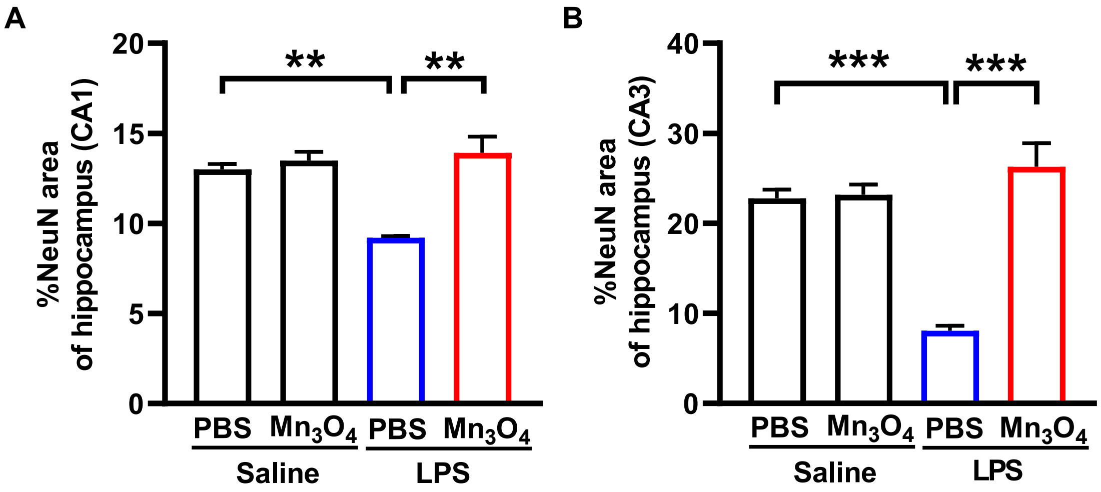

Figure S14. Mn_3O_4 nanozymes alleviated neuronal damage caused by LPS injection into the bilateral hippocampus of C57BL/6J mice. (A-B) Statistics of NeuN-positive areas in CA1 and CA3 regions of the hippocampus of LPS-injected mice treated with Mn_3O_4 nanozymes. Data are presented as mean ± SEM, n = 3. ANOVA was used for multigroup comparisons. $^{***}P<0.001$ and $^{**}P<0.01$.

Supplementary Figure 15

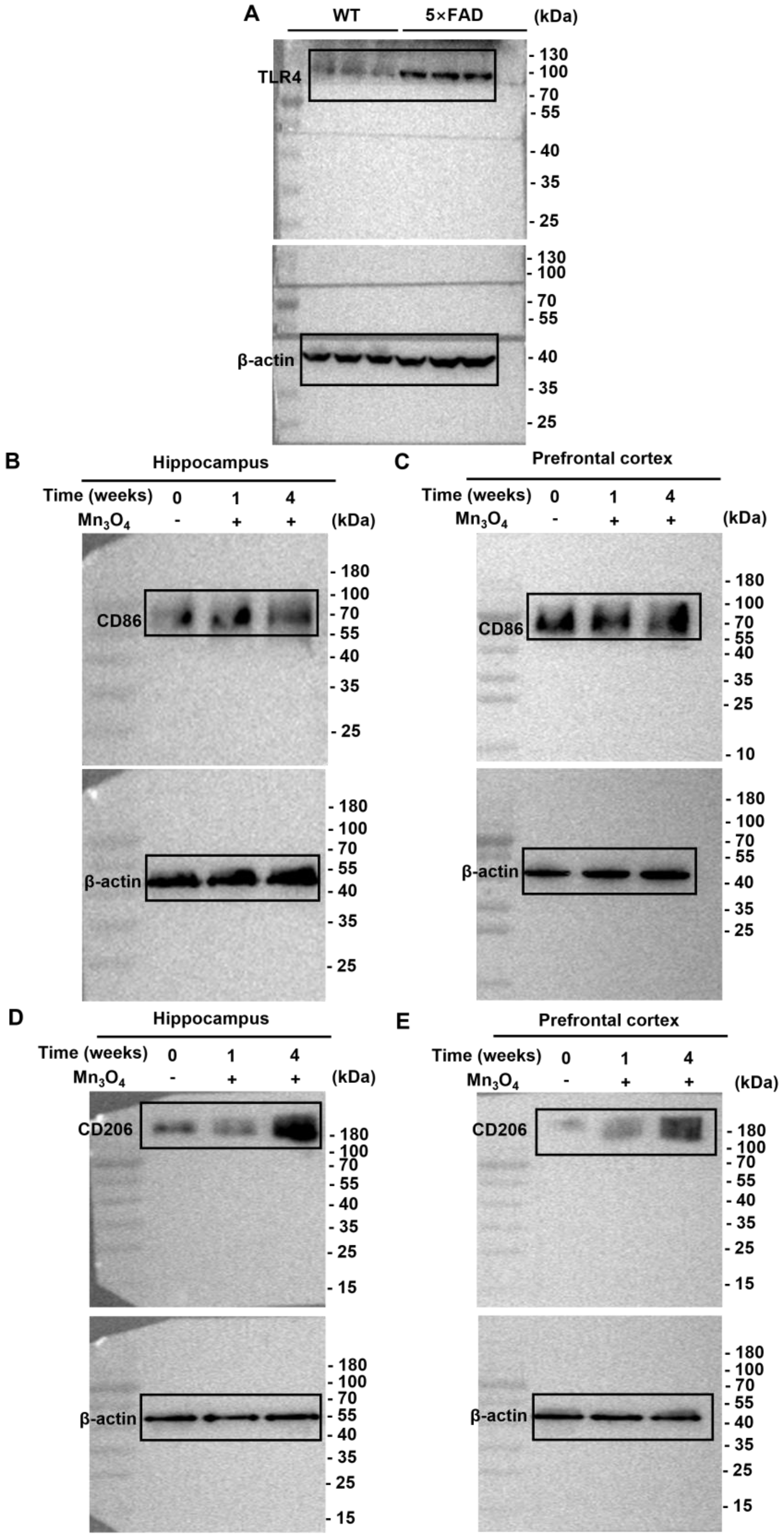

Figure S15. Uncropped western blotting images of Figure 3. (A) Uncropped western blotting images of TLR4 in 5×FAD mice and wild-type mice in Figure 3A. **(B-E)** Uncropped western blotting images of CD86 and CD206 in hippocampal and prefrontal cortical tissues after 1-4 weeks of Mn_3O_4 nanozyme treatment in Figure 3E.

Supplementary Figure 16

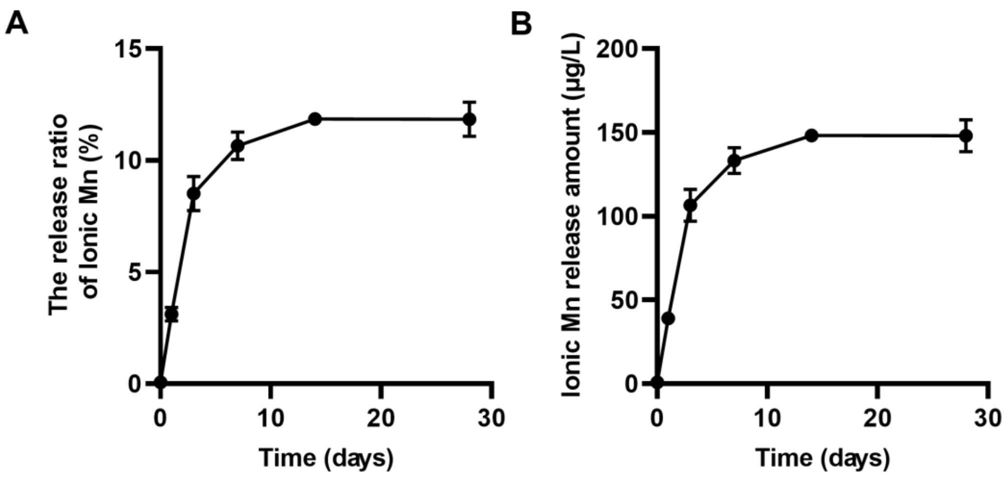

Figure S16. Degradation and release curves of Mn ions by Mn_3O_4 nanozymes in the simulated nasal environment. (A) Mn ion release rate curves of Mn_3O_4 nanozymes placed in simulated nasal electrolyte solution at 37 °C for 28 days. **(B)** Cumulative Mn ion release curve of Mn_3O_4 nanozymes placed in 37 °C electrolyte solution for 28 days, n = 3. Data are presented as mean ± SEM.

Supplementary Figure 17

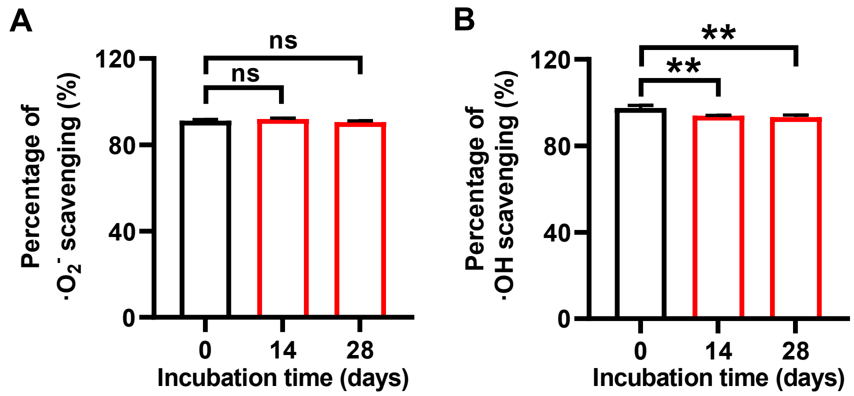

Figure S17. Effect of the stability of Mn_3O_4 nanozymes on their antioxidant activity. (A-B) Changes in the ability of Mn_3O_4 nanozymes to scavenge $\cdot O_2^-$ and ·OH after incubation at 37 °C for 28 days, n = 3. Data are presented as mean ± SEM. ANOVA was used for multigroup comparisons. $^{**}P$<0.01 and ns (no significance).

Supplementary Figure 18

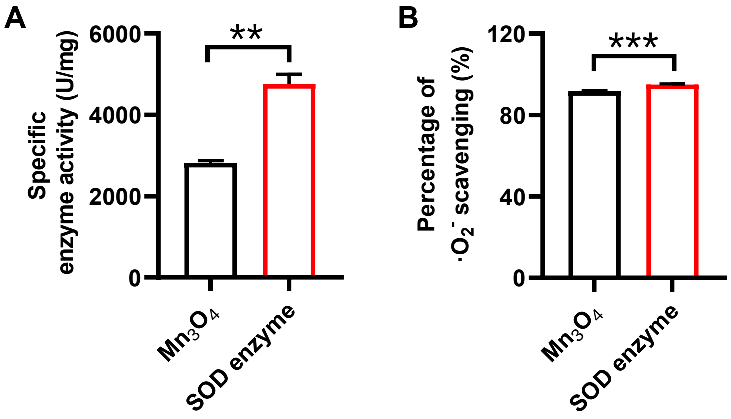

Figure S18. Comparison of SOD-like activity parameters between Mn_3O_4 nanozymes and natural SOD enzymes. (**A**) SOD specific enzyme activity of Mn_3O_4 nanozymes and natural SOD enzymes, n = 3. (**B**) Percentage of $\cdot O_2^-$ scavenging by Mn_3O_4 nanozymes and natural SOD enzymes, n = 3. Data are presented as mean ± SEM. Unpaired t-test was used for two-group comparisons. $^{***}P$<0.001 and $^{**}P$<0.01.

Supplementary Figure 19

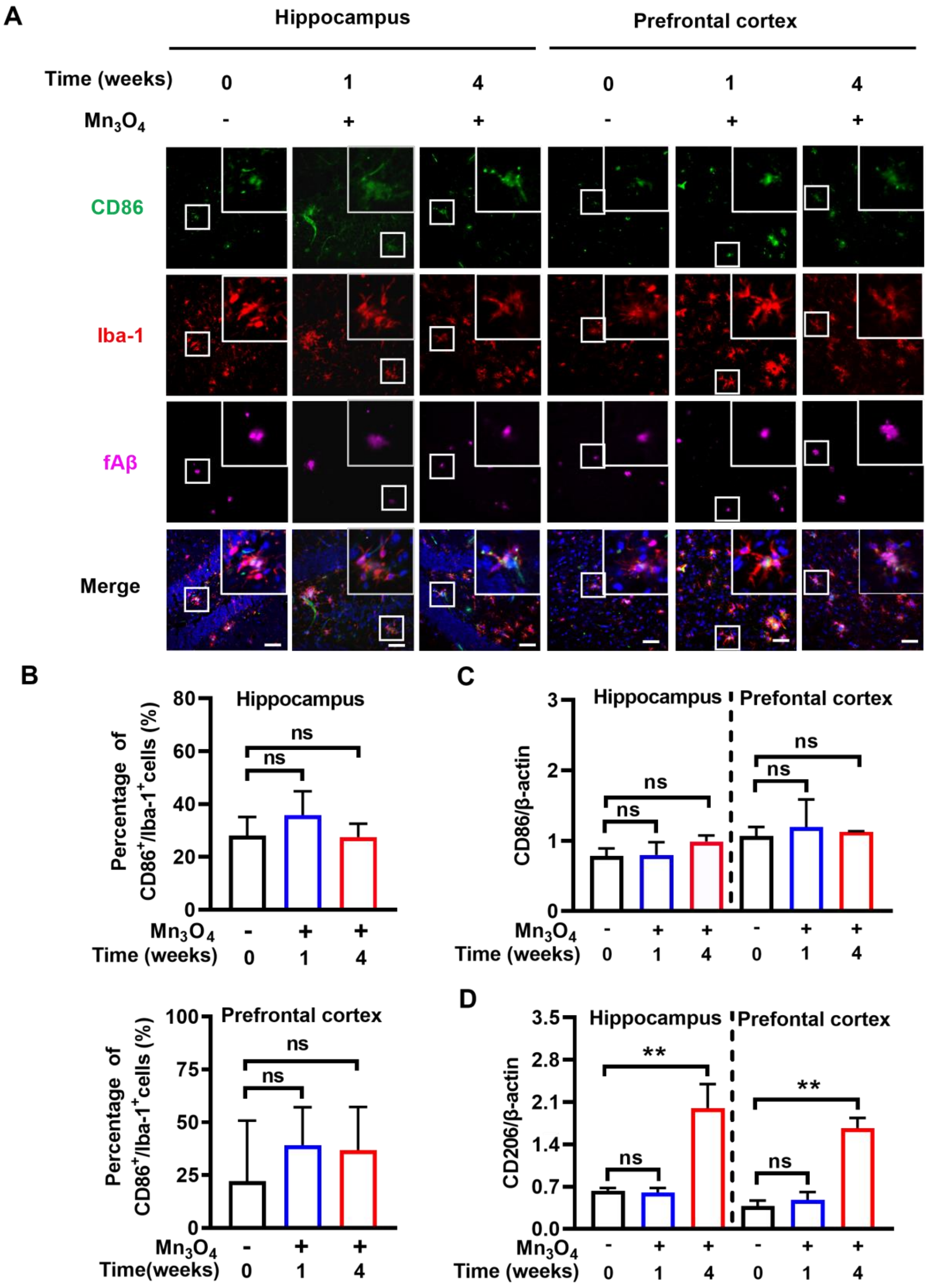

Figure S19. Modulation of microglial phenotype after 1-4 weeks of treatment with Mn_3O_4 nanozymes. (A) Immunofluorescence staining of CD86 (green), Iba-1 (red), and fAβ plaques (magenta) in the DG region of the hippocampus and prefrontal cortex of 5×FAD mice treated with Mn_3O_4 nanozymes for 1-4 weeks, scale bar = 50 μm. Magnified images of the box areas

are shown in the upper right corner of each image. **(B)** Statistics of the percentage of $CD86^+$/$Iba\text{-}1^+$ cells in magnified images of the hippocampus and prefrontal cortex, n = 6. **(C)** Densitometric statistics of CD86 protein expression in hippocampal and prefrontal cortical tissues of 5×FAD mice treated with Mn_3O_4 nanozymes for 1-4 weeks, n = 3. **(D)** Densitometric statistics of CD206 protein expression in the hippocampus and prefrontal cortex tissues of 5×FAD mice after the above treatment, n = 3. Data are presented as means ± SEM. ANOVA was used for multigroup comparisons. $^{**}P<0.01$ and ns (no significance).

Supplementary Figure 20

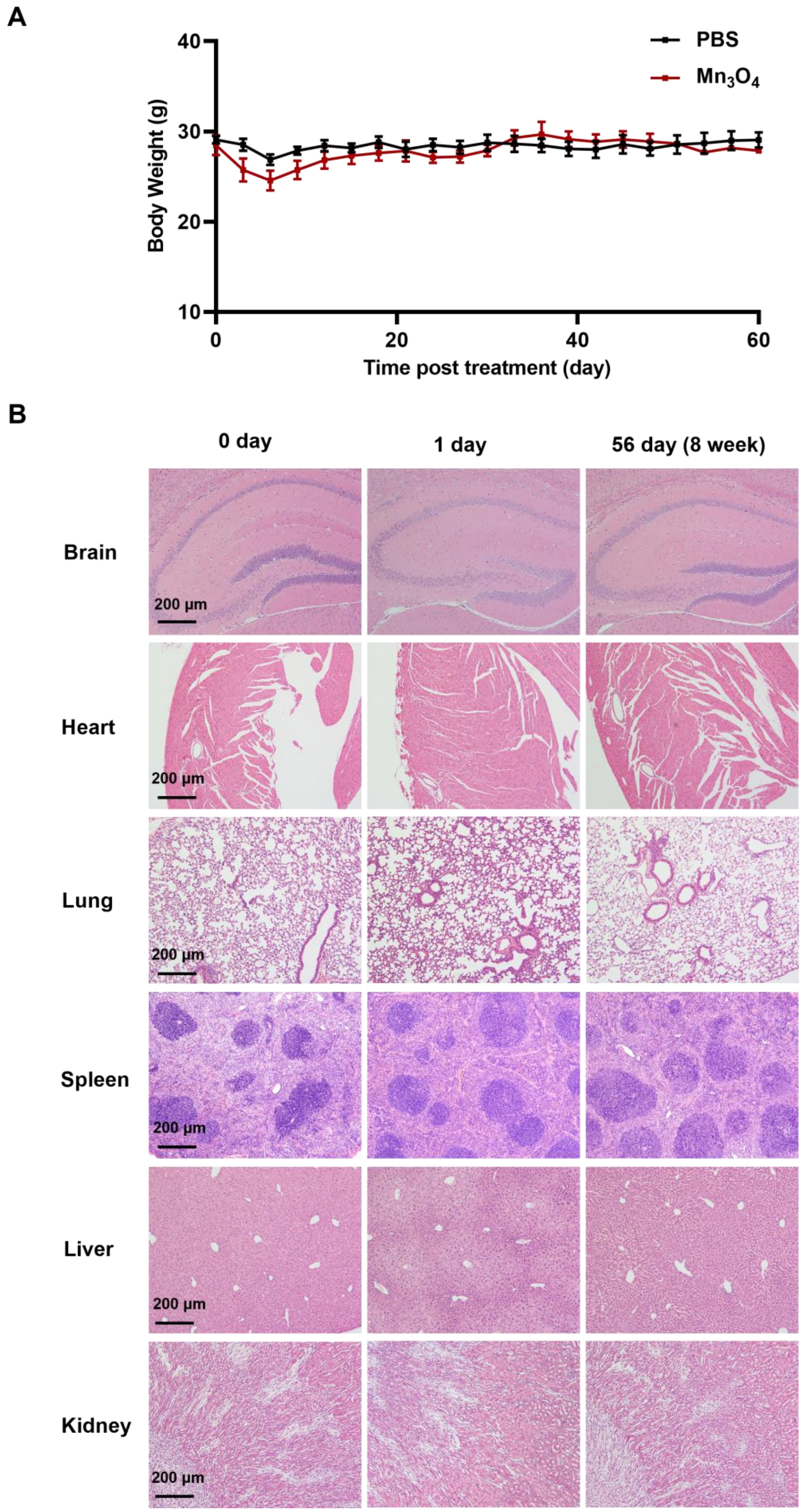

Figure S20. Biosafety evaluation of intranasal administration of Mn_3O_4 nanozymes. (A) Body weight of 5×FAD transgenic mice after 8 weeks of treatment with Mn_3O_4 nanozymes, n = 3-6. Data are presented as mean ± SEM. **(B)** Representative H&E staining images of main organs of mice after 24 h or 8 weeks after intranasal administration of Mn_3O_4 nanozymes, scale bar = 200 μm.

Supplementary Figure 21

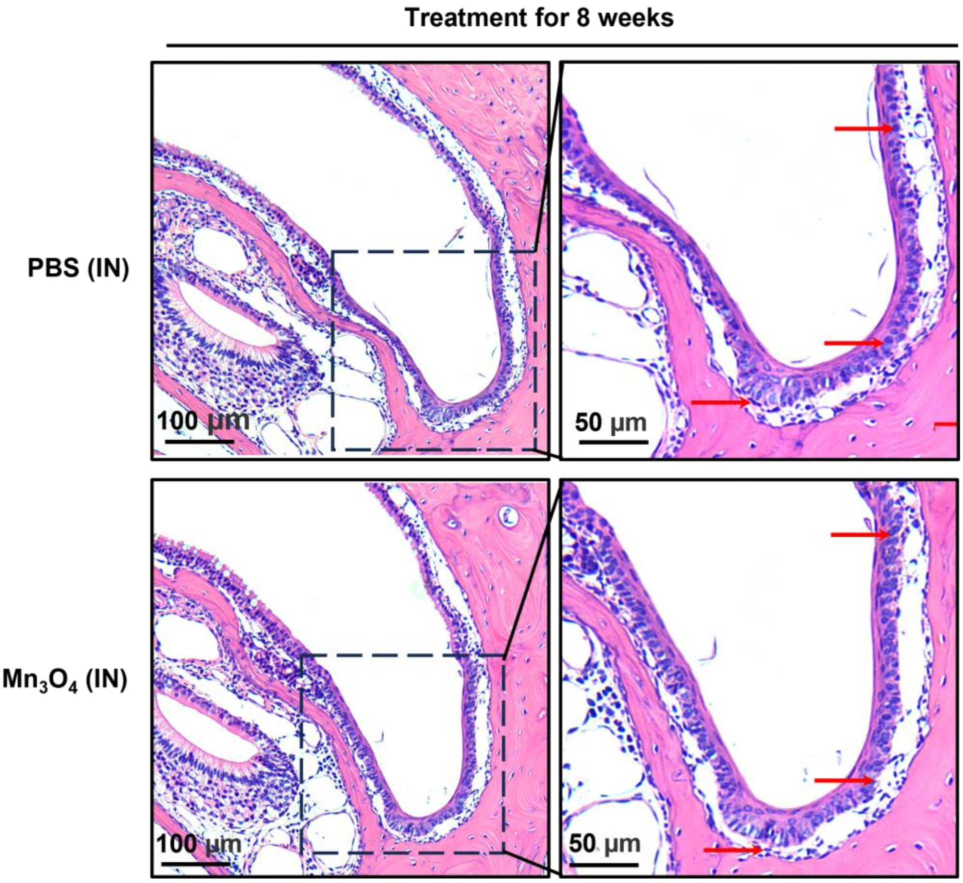

Figure S21. Biosafety assessment of nasal epithelial tissue after 8 weeks of continuous intranasal administration of Mn_3O_4 nanozymes. Representative images of H&E staining of nasal epithelial tissue of C57BL/6J mice after 8 weeks of Mn_3O_4 nanozyme treatment, scale bar = 50 μm.

Supplementary Figure 22

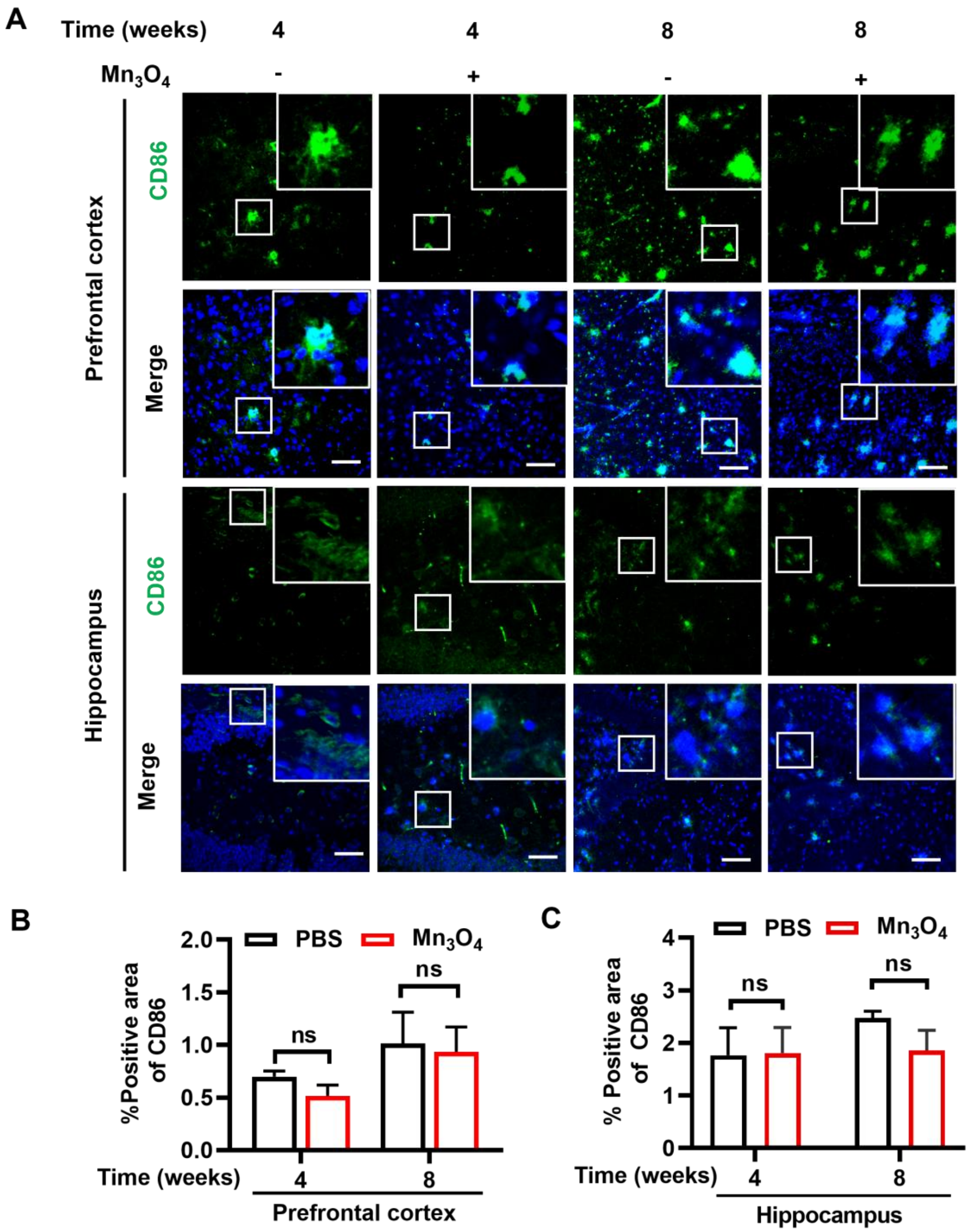

Figure S22. Mn_3O_4 nanozyme treatment for 4 and 8 weeks failed to modulate M1 microglia in 5×FAD mice. (A) Immunofluorescence staining of CD86 protein in the prefrontal cortex and hippocampus after 4 and 8 weeks of Mn_3O_4 nanozyme treatment, scale bar = 50 μm. **(B-C)** Statistical analysis of the percentage of CD86-positive areas in the prefrontal cortex and hippocampus after the indicated treatment. Data are presented as means ± SEM, n = 3. Multiple t-tests were used for multigroup comparisons, ns (no significance).

Supplementary Figure 23

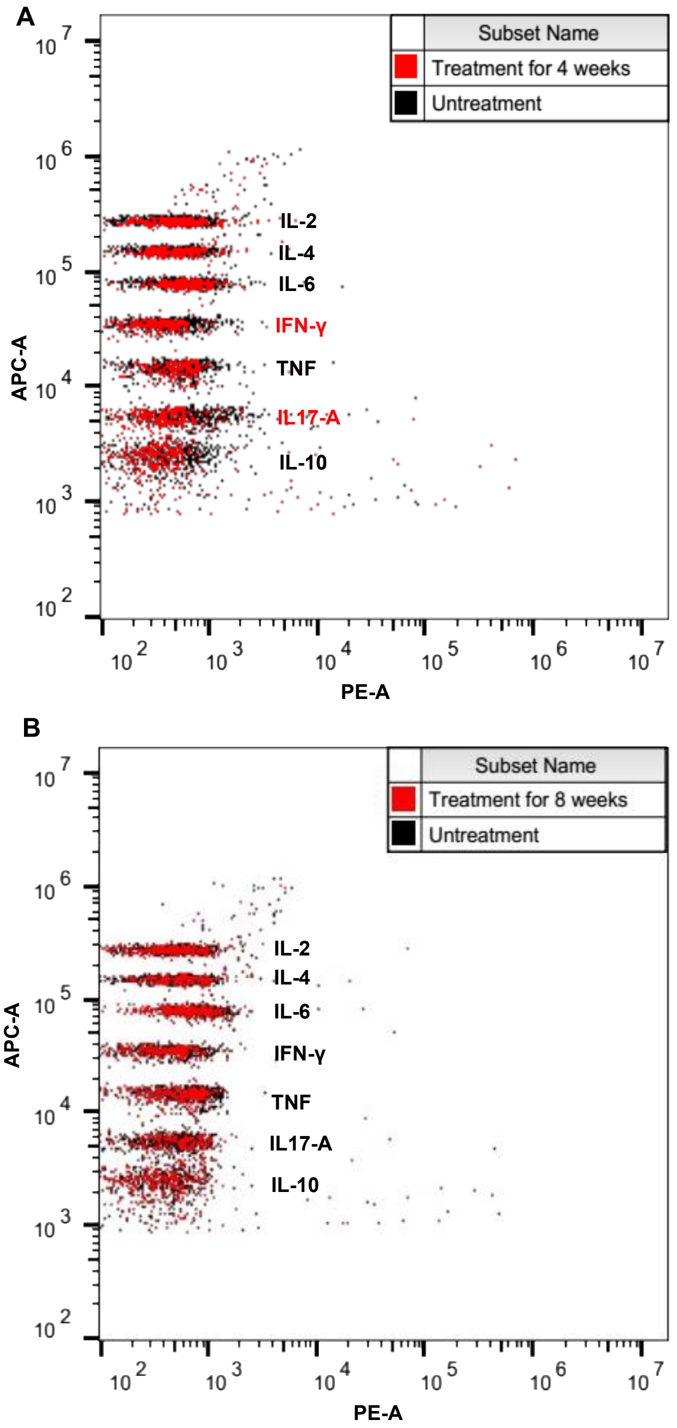

Figure S23. Effects of 4 and 8 weeks of Mn_3O_4 nanozyme treatment on the secretion of serum inflammatory factors in 5×FAD mice. (A) Flow cytometry plots of serum inflammatory factors after 4 weeks of Mn_3O_4 nanozyme treatment. **(B)** Flow cytometry plots of serum inflammatory factors after 8 weeks of corresponding treatments.

Supplementary Figure 24

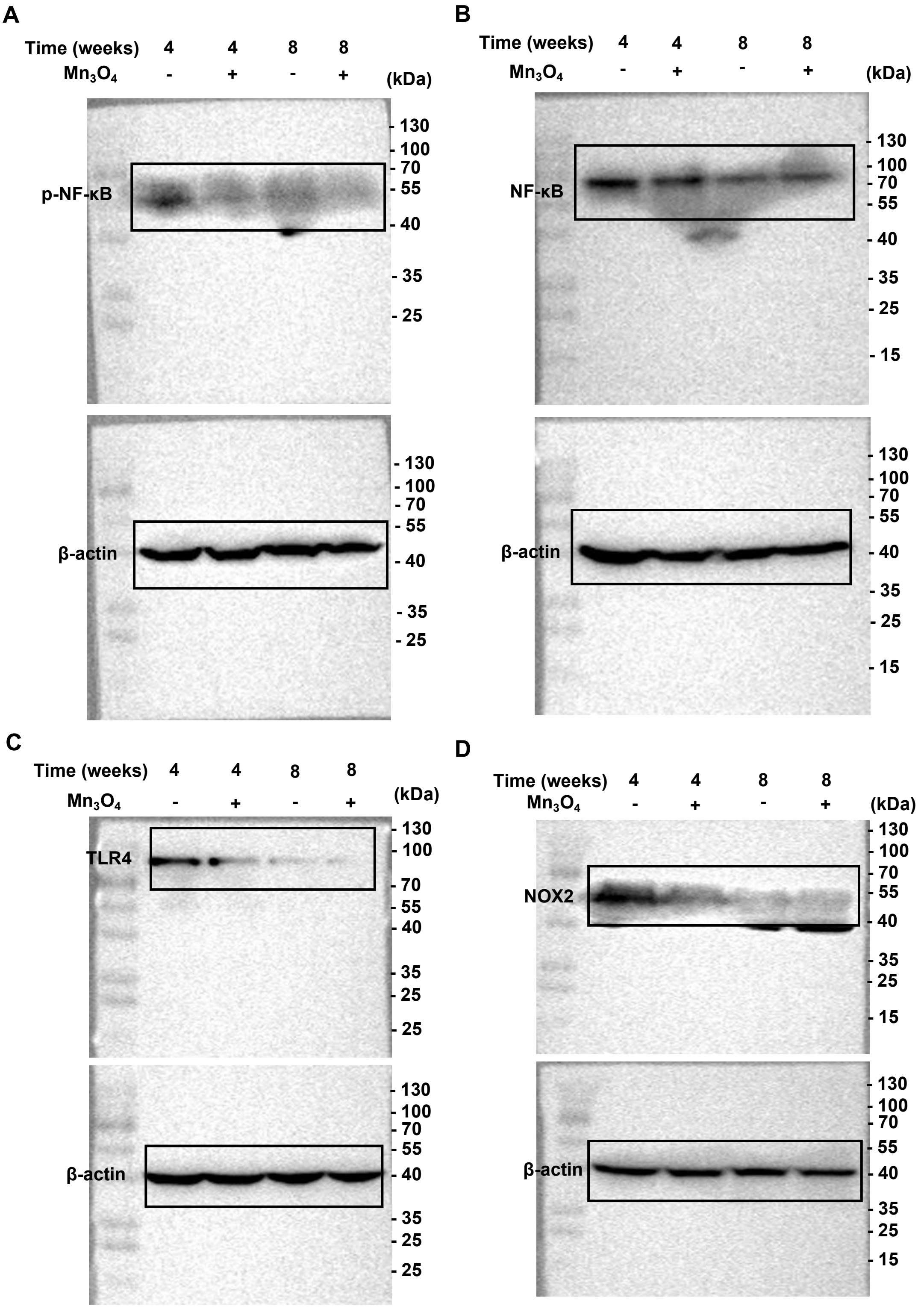

Figure S24. Uncropped western blotting images from Figure 6. (**A-B**) Expression of p-NF-κB and NF-κB in hippocampal and cortical tissues after 4-8 weeks of treatment with Mn_3O_4 nanozymes in 5×FAD mice in Figure 6C. (**C-D**) Expression of TLR4 and NOX2 in hippocampal and cortical tissues after 4-8 weeks of treatment with Mn_3O_4 nanozymes in 5×FAD mice in Figure 6E.

Supplementary Figure 25

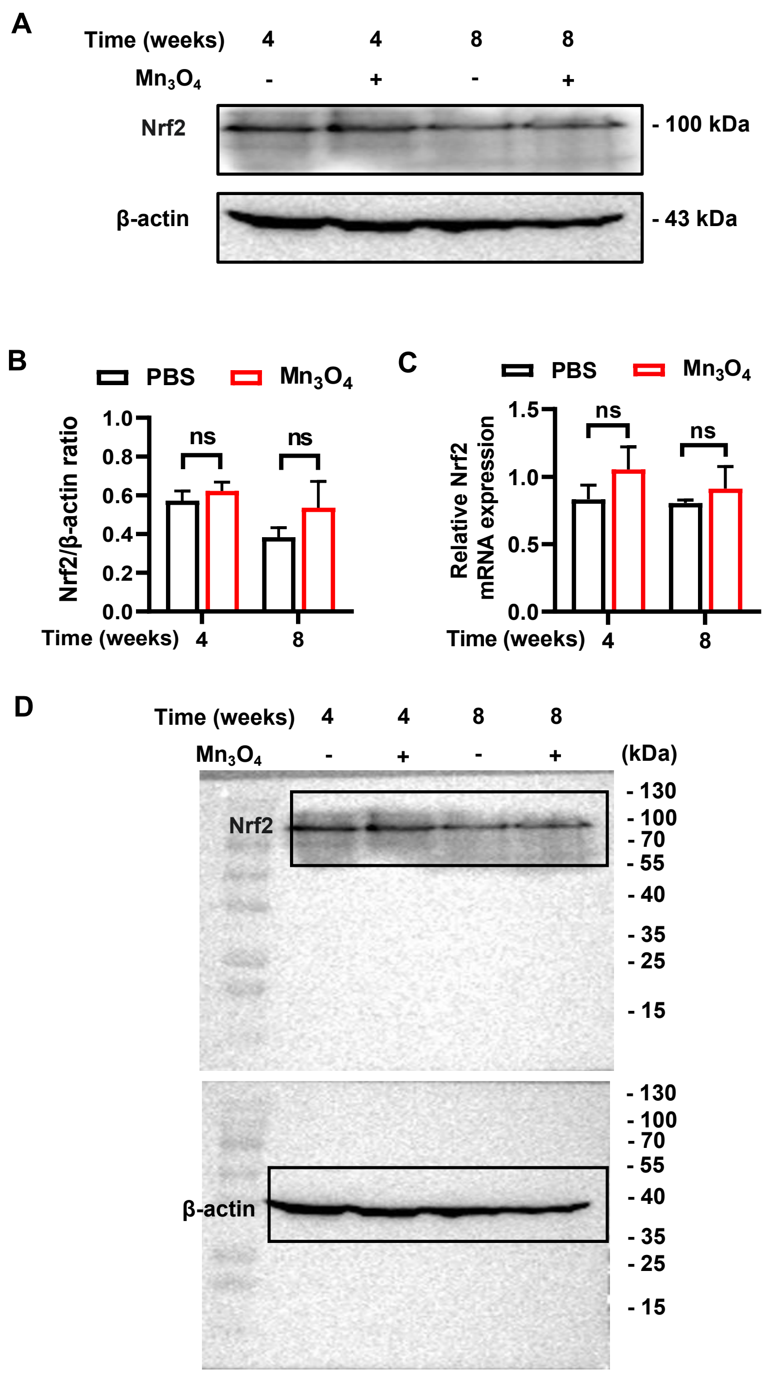

Figure S25. The inhibitory effect of Mn_3O_4 nanozymes on ROS generation was not significantly correlated with the modulation of Nrf2. (A-B) Immunoblotting results and densitometric analysis of Nrf2 in the prefrontal cortex and hippocampus after 4-8 weeks of Mn_3O_4 nanozyme treatment, n = 3 per group. **(C)** Relative mRNA expressions of Nrf2 in the prefrontal cortex and hippocampus after the indicated treatments, n = 4 per group. **(D)** Uncropped western blotting images of Nrf2 in the prefrontal cortex and hippocampus after 4-8 weeks of Mn_3O_4 nanozyme treatment. Data are presented as mean ± SEM. Multiple t-tests were used for multigroup comparisons, ns (no significance).

Supplementary Figure 26

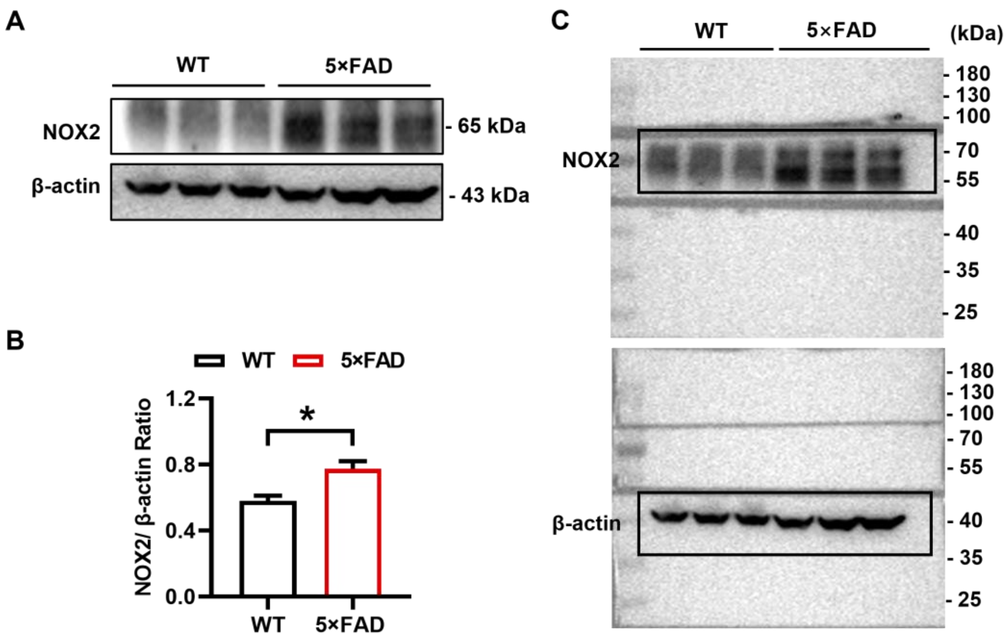

Figure S26. Expression of NOX2 subunits in the prefrontal cortex and hippocampus of wild-type and 5×FAD mice. (A) Immunoblotting results of NOX2 subunits determined by western blotting. **(B)** Densitometric analysis of NOX2 subunits determined by western blotting, n = 4 per group. (**C**) Uncropped western blotting images of NOX2 in 5×FAD mice and wild-type mice. Data are presented as mean ± SEM. Unpaired t-test was used for two-group comparisons. $^{*}p < 0.05$.

Supplementary Figure 27

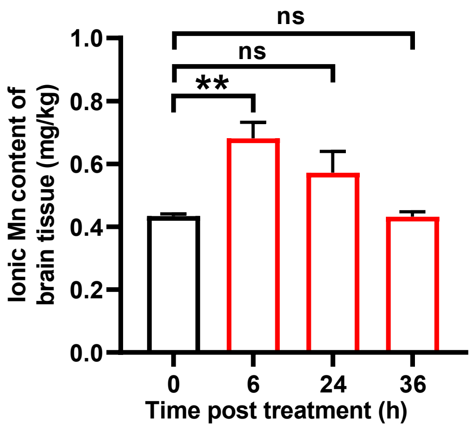

Figure S27. Mn ion metabolism analysis of Mn_3O_4 nanozymes in brain tissue. Statistical analysis of Mn ion content in brain tissue at 6, 24, and 36 h after intranasal administration, n = 7. Data are presented as mean ± SEM. ANOVA was used for multigroup comparisons. $^{**}p < 0.01$, and ns (no significance).
